# Xiao Qing Long Tang ameliorates neutrophil extracellular trap-dendritic cells-T helper 17 cell axis in Neutrophilic Asthma

**DOI:** 10.1371/journal.pone.0336333

**Published:** 2025-11-06

**Authors:** Xiaoying Ji, Hongda Chen, Sheng-dong He, Min Huang, Xiaoli You, Chuan Xiao, Zhifeng Chen, Jinwen Cai

**Affiliations:** 1 Department of Respiratory and Critical Care Medicine, The Affiliated Hospital of Guizhou Medical University, Guiyang, GuizhouChina; 2 Department of Chinese Medicine, The Seventh Affiliated Hospital, Sun Yat-sen University, Shenzhen, Guangdong, China; 3 Department of Respiratory and Critical care Medicine, The First Affiliated Hospital of Guangxi University of Chinese Medicine, Nanning, Guangxi, China; 4 Department of Neurology, The Seventh Affiliated Hospital, Sun Yat-sen University, Shenzhen, China; 5 Department of Critical Care Medicine, the Affiliated Hospital of Guizhou Medical University, Guiyang, Guizhou, China; 6 Department of Respiratory and Critical Care Medicine, The Second Xiangya Hospital, Central South University, Changsha, Hunan, China; 7 Department of Respiratory and Critical Care Medicine, The Third Xiangya Hospital of Central South University, Changsha, Hunan China; Tokyo University of Pharmacy and Life Sciences: Tokyo Yakka Daigaku, JAPAN

## Abstract

**Background:**

Neutrophilic asthma (NA) is an allergic airway inflammation disease featuring heterogeneous neutrophil infiltration, which is driven by the interactions between dendritic cells (DCs) and T helper (Th) 17 cells. Neutrophils release neutrophil extracellular traps (Nets), which promote disease progression and glucocorticoid resistance. Therefore, targeting the interaction among Nets, DC and Th17 is a promising pathway for preventing organ damage. Traditional Chinese Medicine (TCM), especially Xiao-qing-long-tang (XQLT), has shown potential in managing eosinophilic asthma by modulating Th2 cell-mediated inflammation, reducing eosinophilic infiltration, and airway remodeling. However, XQLT’s effect on Nets and DCs-Th17 interactions in NA remains unclear.

**Methods:**

We developed two models: an ovalbumin (OVA)/lipopolysaccharide (LPS)-induced NA mouse model with interventions using either XQLT or sivelestat, and a series of bone marrow-derived dendritic cells (BMDCs)-Th17 cell differentiation models induced by Nets, OVA/LPS, OVA/LPS/Nets, XQLT, OVA/LPS/Nets/XQLT, or corresponding inhibitors. The chemical composition of XQLT was analyzed using ultra-performance liquid chromatography-mass spectrometry (UPLC-MS). Key parameters were evaluated via histopathology, immunohistochemistry, immunofluorescence scanning, flow cytometry, Western blot (WB) analysis, and enzyme-linked immunosorbent assay (ELISA).

**Results:**

In OVA/LPS-induced mice, treatment with sivelestat in OVA/LPS-induced mice reduced airway inflammation, Nets formation characterized by citrullinated histone H3 (CitH3) and myeloperoxidase (MPO) expressions, Th2/17 cell proportions in lungs, and interleukin (IL)-4, 6, 17, and 23 levels in bronchoalveolar lavage fluid (BALF). In vitro, OVA/LPS/Nets promoted IL-6/23 secretions and Th17 differentiation through increased p38 mitogen-activated protein kinase (MAPK)/nuclear factor κB (NF-κB) signaling phosphorylation in DCs. Fifty-one compounds were identified in XQLT, with 11 predicted to bind MAPK proteins with high affinity. XQLT significantly inhibited Nets-DCs-Th17 Axis and p38MAPK/NF-κB signaling in both NA mouse and cell models.

**Conclusion:**

XQLT offered a promising treatment strategy for regulating the Nets-DCs-Th17 axis in NA.

## Introduction

Neutrophilic asthma is a Th17-associated asthma phenotype characterized by a significant presence of neutrophils (>50%) and minimal eosinophils (<2%) in induced sputum [1–3]. Although only 10% of all asthmatic patients, were characterized by airway neutrophil-predominant inflammation and responded poorly to conventional asthma therapies, this subgroup accounted for over 60% of asthma-related healthcare costs [4,5]. The excessive neutrophil recruitment in NA resulted from a complex cascade of amplification mechanisms. Accumulating evidence suggested the inflammatory imbalance induced by the DCs - Th17 axis played a role in the progression of NA. Allergens and pathogens activated DCs, which cooperated with effector Th17 cells to secrete IL–17. In turn IL-17 prompted airway epithelial cells to secrete neutrophilic attracting chemokines like IL – 8 and CXCL8 for recruiting and activating neutrophils [6–10]. Elevated levels of IL-6 from DCs facilitated the initial formation of Th17 cells, while IL-23 from DCs supported their expansion and survival [11–13]. Collectively, DCs responded to local microenvironmental cues and induced specific Th17 cell subsets by releasing polarized cytokines IL-6 and 23 in NA [14,15].

Neutrophils, typically regarded as terminal effector cells, combat pathogens and drive inflammation through mechanisms such as phagocytosis, reactive oxygen species production, and degranulation. Interestingly, clinical studies have found that activated neutrophils further released Nets, which played immunostimulatory roles and exacerbated airway inflammation in NA. Increased Nets structures and Nets-associated proteins, including double-strand DNA (dsDNA), myeloperoxidase (MPO)-DNA complexes, and CitH3, were associated with poor symptom control, corticosteroid resistance, and impaired lung function in NA patients [1,16,17]. BALF containing Nets from a Sendai virus-induced asthma model directly stimulated DCs to release a substantial amount of IL-6 [18]. Targeting Nets has been shown to improve airway neutrophilic inflammation and mitigate steroid resistance [18,19]. Recent study also found that Nets affected bronchial epithelial cells and triggered a second wave of neutrophil inflammation amplification by secreting chemokines in NA [20]. As recently recognized, Nets were highly effective in forming self – sustained vicious cycles to amplify the inflammatory response in NA. These studies indicated that an interaction between Nets and DCs-Th17 axis in the pathogenesis of NA, However, the precise mechanisms remained inconclusive.

TCM has been integrated into asthma treatment protocols in China and showed promise as an immunomodulator for improving asthma symptoms. Among these TCM for eosinophilic asthma treatment, XQLT was a well-established prescription known for its ability to effectively reduce Th2 cytokine levels by targeting the thymic stromal lymphopoietin-NF-κB pathway [21]. Additionally, XQLT has been shown to reverse Th2 cell imbalance by inhibiting DC activation [22]. However, TCM laid in its ability to provide treatment based on syndrome differentiation, not inflammation phenotype. There was still a lack of research on whether TCM can exert unique therapeutic effects on different phenotypes of the same disease. Therefore, it remained uncertain whether XQLT suppressed the Nets - DCs - Th17 axis in NA.

To address this gap, our study hypothesized that XQLT played a protective role in mitigating Nets-DCs-Th17 axis in NA. To test our hypothesis, we first evaluated the effects of Nets on DC-mediated Th17 cell differentiation in NA mouse and cell models. Subsequently, we analyzed the chemical composition of XQLT using UPLC-MS and speculate their potential drug targets by molecular docking. Finally, we investigated the impact of XQLT on NA mouse and cell models to elucidate its potential therapeutic mechanism in NA.

## Materials and methods

### Preparation and characterization of XQLT

The herbal concentrate granules of XQLT were provided by the Affiliated Hospital of Guizhou Medical University (Guizhou, China) and authenticated by Professor Hongda Chen. According to the Chinese Pharmacopoeia Commission (2020), XQLT was composed of Paeoniae Radix Alba (Baishao), Pinelliae Rhizoma Praeparatum (Fa Banxia), Zingiberis Rhizoma Recens (Ganjiang), Cinnamomi Ramulus (Guizhi), Ephedrae Herba (Mahuang), Schisandrae Fructus (Wuweizi), Asari Radix et Rhizoma (Xixin), and Glycyrrhizae Radix et Rhizoma Praeparata (Zhi Gancao). The botanical identities of these herbs were verified using the plantlist package in R (version 3.6.2) and cross-referenced with data from The Plant List (http://www.theplantlist.org). Detailed herb compositions were available in Supplementary S1 Table.

XQLT was prepared according to established procedures as previously described [23]. Briefly, the herbs with corresponding weights in Supplementary S1 Table were macerated in 600 ml of distilled water, brought to a boil, stirred and filtered. Then, the obtained 270 ml of the medicinal liquid was concentrated to a final concentration of 0.167 g/ml. The main constituents of XQLT were analyzed using ultra-performance liquid chromatography-quadrupole/ orbitrap high-resolution mass spectrometry (UPLC-Q Exactive Focus HRMS, Thermo Fisher Scientific, USA). All standards were prepared by dissolving them in methanol, yielding a final concentration of 10 µg/ml. The detailed UPLC-MS parameters and standards are provided in Supplementary Material S2 Table.

### Animal experiment design

Male specific pathogen-free (SPF) BALB/c mice (6–8 weeks old, 18–20 g) were purchased from Hunan SJA Experimental Animal Company (Changsha, China, certificate number: 430727230103254647). Mice were housed under SPF conditions with a 12-hour light/dark cycle, controlled temperature (22 ± 4°C), and humidity (40–60%). Ethical approval was obtained from the Ethics Board of the Affiliated Hospital of Guizhou Medical University (approval code: 2101251). For our study, clinical trial registration was not applicable.

**Fig 1:**
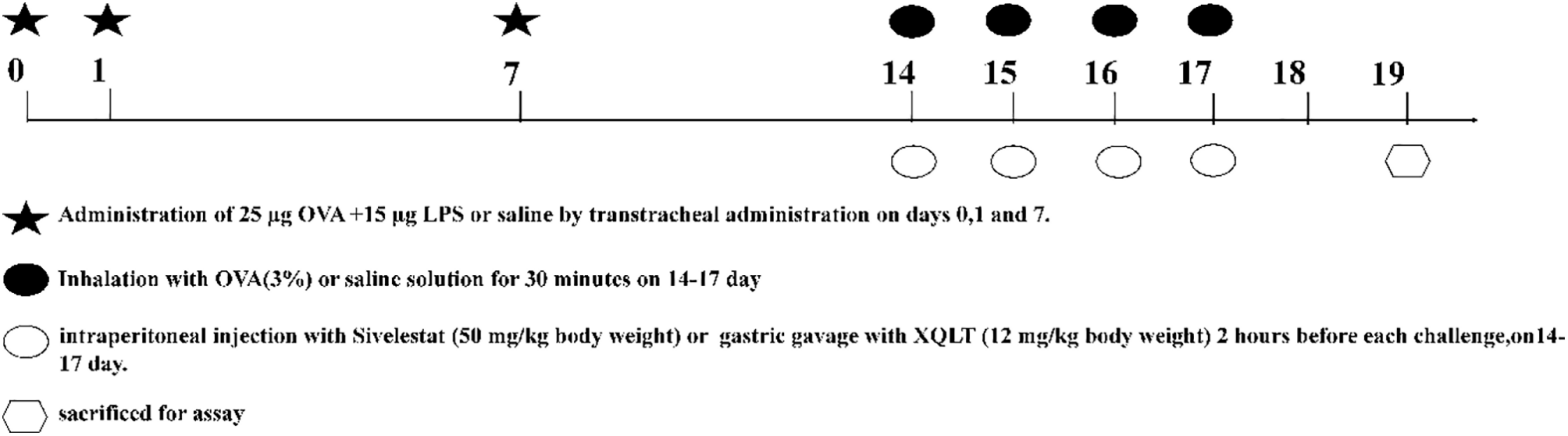
The schematic experimental procedure of neutrophilic asthma mouse models.

The experimental protocols followed established methods (Fig 1) [24]. Using the numerical table method, the mice were assigned to six groups (n = 3 per group): control (con), XQLT, Sivelestat (MCE, USA, HY-17443) (SIV), OVA (SIGMA, USA, A5503)/LPS (SIGMA, USA, L2630) (OL), OVA/LPS/Sivelestat (OLS), and OVA/LPS/XQLT (OLX). In the OL group, sensitization was performed via intratracheal injections of 25 µg OVA and 10 µg LPS (in 40 µL saline) on days 0, 1, and 7, followed by aerosolized 3% OVA challenge on days 14–17 [24]. The con group received sensitization and challenge with 0.9% saline at the same time points. In the OLS or OLX groups, Sivelestat (50 mg/kg body weight) [25] or XQLT (12 mg/kg body weight) [23] was administered intraperitoneally or with gastric gavage respectively, 2 hours before each challenge, following the OL group protocol. In the XQLT or SIV groups, the mice received treatment at the same time as the OLX or OLS groups, respectively, based on the con group protocol. Forty-eight hours after the final challenge, mice were euthanized with intraperitoneal pentobarbital, and samples were collected for analysis. No mortality occurred during the experiment.

### Isolation and culturing of cells

BMDCs and neutrophils were obtained and differentiated from the bone marrow of naïve 6–8-week-old BALB/c mice via density gradient centrifugation, following established protocols [26]. CD4 + T lymphocytes were isolated from spleen cells using a protocol adapted from [27].

Bone marrow cells were harvested from the femurs and tibias and subjected to centrifugation at 1200 rpm for 5 minutes at 4°C. Erythrocytes were lysed using an erythrocyte lysis buffer (Abiowell, China, AWC0390). The remaining cells were cultured in 6-well plates(Corning, USA, 3516) at a density of 1 × 10^6^ cells/mL in RPMI medium (Abiowell, China, AWM002), supplemented with murine granulocyte-macrophage colony-stimulating factor (GM-CSF) (MCE, USA, HY-P7361) (20 ng/mL) and IL-4(MCE, USA, HY-P70644) (20 ng/mL), for 6 days. Cytokines were refreshed, and half of the medium was replaced every 2 days to promote the differentiation of BMDCs. The purity of BMDCs (>85%) was confirmed by flow cytometry based on CD11c and MHCII expression. Neutrophils were isolated using a mouse bone marrow neutrophil isolation kit (Solarbio, china,

P8550) followed by density gradient centrifugation, and purity (>95%) was confirmed by Giemsa staining. CD4 + T lymphocytes were isolated from spleen cell suspensions using magnetic bead separation (Miltenyi Biotec, Germany 130-117-043,).

### Induction and quantification of nets

Nets were induced as previously described [28]. Isolated neutrophils were seeded at a density of 1 × 10^6^ cells/ml in 12-well cell culture plates with serum-free RPMI 1640 medium to allow for cell adhesion. Subsequently, the neutrophils were stimulated with 500 nM Phorbol 12-myristate13-acetate (PMA) (SIGMA, USA, P1585) for 6 hours. After incubation, the culture medium was removed, and the Nets layer was scraped from the plates. The plates were then rinsed three times with phosphate-buffered saline (PBS), and the collected solution was centrifuged at 500 g for 5 minutes at 4°C to remove neutrophils, yielding a supernatant enriched with Nets. Given that Nets were composed of antimicrobial proteins and double-stranded DNA, the concentration of dsDNA was measured with the Mouse dsDNA ELISA Kit (mlbio, China, ml058205). The concentration of the obtained supernatant was determined to be 13.92 ug/ml. Finally, the supernatant was resuspended in cold PBS for subsequent experiments.

### Th17 cells differentiation induced by BMDCs in the presence of different stimulus

To assess the impact of Nets-BMDCs stimulation on Th17 cell differentiation, BMDCs were plated in 12-well plates at a concentration of 1 × 10^6^ cells/mL and exposed to the following conditions for 48 hours: Nets(5 µg/ml) [29], OL (100 µg/ml OVA + 100 ng/ml LPS), OLN (100 µg/ml OVA + 100 ng/ml LPS + 5 µg/ml Nets), XQLT(100 µg/ml), OLNX (100 µg/ml OVA + 100 ng/ml LPS + 5 µg/ml Nets+100 µg/ml XQLT) or left untreated as a control. After stimulation, BMDCs were cleaned with PBS containing 0.5% bovine serum albumin (BSA) and then combined in co-culture with spleen-derived CD4 + T lymphocytes at a 1:5 ratio for 3 days, following a previously described protocol [30].

To explore the signaling pathways involved in OVA/LPS/Nets-induced BMDCs in regulating Th17 cell differentiation, BMDCs were pre-treated with specific inhibitors: 10 µM p38-MAPK specific inhibitor SB202190(MCE, USA, HY-10295) and 5 µg/ml NF-κB-specific inhibitor DHMEQ (MCE, USA, HY-14645) 1 hour before being stimulated with OVA/LPS/Nets. The stimulated BMDCs were then washed with PBS containing 0.5% BSA and co-cultured with CD4 + T lymphocytes.

After stimulation, the relevant proteins expression in signaling pathways and cytokines in the culture medium supernatants were assessed. On day 4 of co-culture, cells were stained for flow cytometry analysis to evaluate Th17 cell differentiation. The supernatants from the cell culture were collected to measure cytokine secretion. Each experiment was repeated three times.

### Cell count in BALF

BALF collection was conducted following established protocols [31]. Briefly, 1 ml of cold PBS with 1 mM Ethylene Diamine Tetraacetic Acid (EDTA) and 2% fetal bovine serum (FBS) was injected into mouse lungs, followed by gentle aspiration to obtain BALF. The collected BALF was centrifuged at 500g for 5 minutes at 4°C, and the supernatants were stored at −80°C for cytokine profile assessment. The isolated cell pellets were resuspended in PBS, centrifuged onto slides, and stained with Wright-Giemsa stain for counting the differential cell types.

### Enzyme-linked immunosorbent assay

The concentrations of IL-4 (Proteintech, USA, KE10010), IL-6 (Proteintech, USA, KE10007), IL-17 (Proteintech, USA, KE10020), and IL-23 (CUSABIO, China, CSB-E08463m) in BALF and cell culture medium were determined using commercial mouse ELISA kits, according to the manufacturer’s recommendations.

### Lung histopathological examination, immunohistochemistry, and immunofluorescence staining

After BALF collection, the right lung tissue of mice was fixed in 4% paraformaldehyde for 24 hours and embedded in paraffin for slicing into 5 μm sections. These slices were stained with hematoxylin and eosin (H&E) or periodic acid–Schiff (PAS) to evaluate lung inflammation and mucus production.

For immunohistochemistry, levels of eosinophil cationic protein (ECP) and Gr-1 were used to evaluate eosinophilic and neutrophilic inflammation in the lung. The sections were first deparaffinized and subjected to antigen retrieval in an antigen repair solution (abiowell, china, AWI0113a). They were then boiled for 20 min, cooled, and washed with PBS. Endogenous peroxidase activity was inhibited using a 1% periodate solution and nonspecific binding was blocked by incubating with 10% goat serum. The tissue sections were then incubated overnight at 4°C with primary antibodies against Gr – 1 (Abcam, UK, ab238132) at a dilution of 1:200 or ECP (Thermo Fisher, USA, PA5–79927) at a dilution of 1:200. Subsequently, the sections were incubated with a secondary antibody for 30 minutes at room temperature. The sections were treated with 3,3 diaminobenzidine, (DAB) and counterstained with haematoxylin.

For immunofluorescence staining of CD11c(Abcam, UK, ab219799), Cit-H3(Abcam, UK, ab281584), MPO (Thermo Fisher, USA, PA5−16672), phospho-p38 MAPK(Abcam,UK, ab4822)(p-p38), phospho-p65 p65(Abcam, UK, ab86299) (p-p65), and phosphor-IkBa (Abiowell, China, AWA44613)(p-IkBa) in lung tissue, the paraffin-embedded sections were deparaffinized and underwent antigen retrieval. To inhibit non-specific binding, the sections were treated with 10% goat serum at 37°C for 30 minutes, and then incubated with the following primary antibodies: anti-CD11c (1:100), anti-CitH3 (1:200), anti-MPO (1:100), anti-p-p38 (1:100), anti-p-p65 (1:100), and anti-p-IkBa (1:100) overnight at 4°C. After washing, the sections were incubated with secondary antibodies for 1 hour at 37°C in the dark. Nuclei were stained using 4,6-diamidino-2-phenylindole (DAPI). Nets were identified by colocalization of MPO, CitH3, and DAPI, while p-p38, p-p65, and p-IkBa in CD11c were identified by colocalization of respective antibodies with DAPI and CD11c. All microscopic images were captured at 400 × magnification using a BA410E microscope (Motic, China).

### Flow cytometry

The minced tissue was digested with collagenase, and the resulting cell suspension was then filtered, centrifuged, and resuspended in Dulbecco’s Modified Eagle Medium containing 2% fetal bovine serum to obtain a single-cell suspension for evaluating the percentage of CD11c⁺MHCII⁺IL-6+ or IL-23 + DCs in lung tissue. Cells were initially incubated with leukocyte activation cocktail (ebioscience, USA, 00-4975-93) at 37°C for 4 hours. Subsequently, the cells were incubated with fluorochrome-tagged monoclonal antibodies against CD11c (Biolegend, USA, 117318) and MHCII (BD, USA, 562367) for 30 minutes at 4°C in a dark environment and then fixed and permeabilized using intracellular fixation and permeabilization buffers (ebioscience, USA, 00-5523-00). After permeabilization, cells were stained with intracellular antibodies against IL-6(eBioscience, USA, 12-7061-82) and IL-23(eBioscience, USA, 45-7123-82) in permeabilization buffer at 4°C in the dark for 30 minutes.

To evaluate the induced percentage of Th2 or 17, cells were first incubated with Cell Stimulation Cocktail (ebioscience, USA, 00-4975-93) at 37°C for 4 hours. Then, cells were incubated with surface markers against CD4 followed by fixation and permeabilization using Intracellular Fixation and Permeabilization Buffer (ebioscience, USA, 00-5523-00). Following this, cells were stained for intracellular markers with intracellular antibodies against IL-4(eBioscience, USA, 12-7041-81) or IL-17(Biolegend, USA, 506916) in permeabilization buffer. Isotype controls were incorporated into the control group, and the results were analyzed using CytExpert software.

### Western blot analysis

Cells were lysed using radio immunoprecipitation assay buffer to prepare protein lysates. The protein concentration of the resulting whole cell lysates was determined with a bicinchoninic acid protein assay kit. Proteins were separated by 10% sodium dodecyl sulfatepolyacrylamide electrophoresis (SDS-PAGE) gels and transferred to a polyvinylidene fluoride (PVDF) membrane. The PVDF membrane was blocked with defatted milk for 90 minutes and then incubated overnight at 4°C with primary antibodies at the following dilutions: p38(Abcam, UK, ab32142) (1:5000), p-p38(Proteintech, USA, 28796–1-AP) (1:4000), IκBα(CST, USA,4814s)(1:1000), p-IκBα(CST, USA,2859s)(1:1000), p65(Proteintech, USA, 10745–1-AP)(1:5000), p-p65(Abiowell, China, AWA47474)(1:5000)). After washing, the membrane was further incubated with secondary antibodies for 90 minutes. β-actin antibody (Proteintech, USA, 66009–1-Ig) (1:5000) served as the loading control. Protein bands were detected using ECL reagent, and ImageJ software was used for quantification, normalizing target protein bands to β-actin.

### Molecular docking

To examine the interaction between essential compounds and primary targets in Nets-stimulated BMDCs in the presence of OVA and LPS, molecular docking techniques were utilized. The core target MAPK14 (human, UniProt P49137) and 11 compounds with the highest Area values from Supplementary Material S3 Table of XQLT were used as receptors and ligands, respectively. The compounds studied in XQLT included albiflorin, cinnamaldehyde, ephedrine, gallic acid, glycyrrhizin, liquiritin, ononin, peoniflorin, pseudoephedrine, schisandrin A, and schisandrol A. Initially, the structures of the target proteins and ligands were obtained from PubChem (https://pubchem.ncbi.nlm.nih.gov/) and the Protein Data Bank (PDB) website (https://www.rcsb.org/). Using PyMOL software, water molecules and small-molecule ligands were eliminated. The functional pockets of the protein receptors were determined based on known ligands from the PDB to aid in the docking process. PyMOL was used for visualizing the docking, and AutoDock Vina for evaluating the binding energies, which were key indicators of the docking outcomes. Binding energies less than −5.00 kcal/mol suggested good binding affinity, while those below −7.00 kcal/mol indicated strong binding affinity.

### Statistical analysis

Our research was a hypothesis-generating study. Statistical analyses were performed using SPSS 26.0 (IBM Corp., Armonk, NY, USA) and Prism 10. Data from three independent experiments were expressed as the mean ± standard deviation (SD). For comparisons involving three groups, statistical differences were calculated by one‐way analysis of variance (ANOVA) with Bonferroni adjustment between multiple groups. Statistical significance was set at a two-tailed p-value < 0.05.

## Result

### Nets removal with Sivelestat inhibited lung inflammation in NA mice models

**Fig 2:**
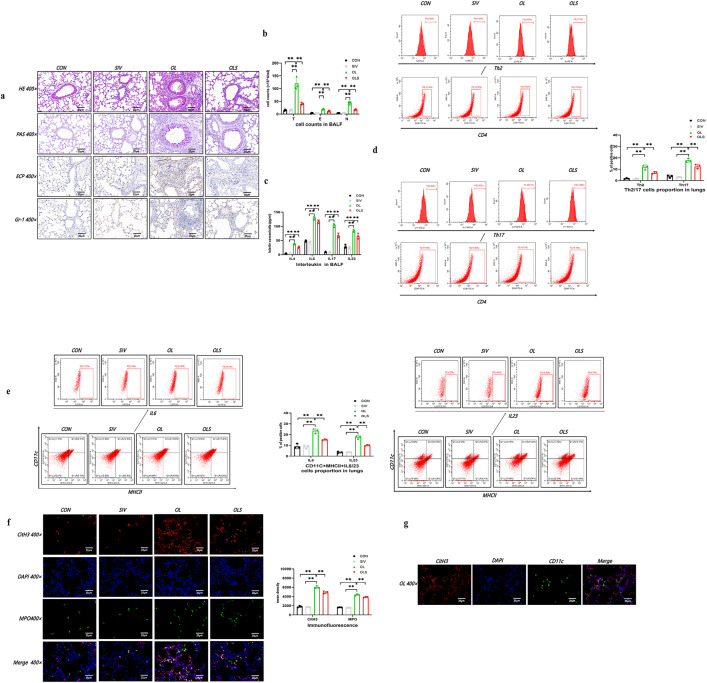
Sivelestat reduced airway inflammation, Nets formation, DC activation and Th differentiation in OVA/LPS-induced neutrophilic asthma models. a. Histological images of lung sections stained with haematoxylin and eosin (H&E), periodic acid–Schiff (PAS), immunohistochemistry for ECP and Gr-1 expression. b. Comparisons of total cell counts (T), eosinophil counts (E), and neutrophil counts (N) (10^4^ cells/ml) in BALF across each group. c. Comparisons of concentrations of IL-4, IL-17, IL-6, and IL-23 in each group. d. Representative flow cytometric analysis and comparisons of Th2/17 cells in lung tissues of each group. e. Representative flow cytometric analysis and comparisons of CD11c+MHC-II + IL-6/23 DCs in lung tissues of each group. f. Identification and comparisons of Nets in lung tissues co-stained with DAPI (blue), cit-Histone3 (red), and MPO (green) using confocal microscopy. g. Immunofluorescence for citH3 and CD11c in the lung of OVA/LPS induced mice (Data are means ± SEM (n = 3); ** P < 0.01) (CON: control group, SIV: Sivelestat group, OL: OVA/LPS group, OLS: OVA/LPS/Sivelestat group).

Firstly, we utilized OVA and LPS co-exposure to induce a NA mouse model [24]. Lung histological analysis with HE and PAS staining found that compared to the control group, the OVA/ LPS group showed significant inflammation, accompanied by goblet cell hyperplasia, (Fig 2a). Immunohistochemistry staining with eosinophilic markers (ECP) and neutrophilic markers (Gr-1) also supported a significant accumulation of eosinophil and neutrophils adjacent to the broncho-vascular bundle in mice treated with OVA/LPS group (Fig 2a). Consistent with the above evaluation, mice induced with OVA/LPS showed increased total cell, neutrophilic and eosinophilic counts in BALF, as compared to the control group, (Fig 2b). Most importantly, the proportion of Th2/Th17 cells and the levels of IL-6 and IL-23 in DCs were significantly higher in the lungs of OVA/LPS-induced mice as well as elevated IL-4, 6, 17, and 23 concentrations in BALF (Fig 2c-e). Overall, these outcomes indicated that we successfully establish pathological features of neutrophilic asthma mouse model, characterized by dendritic cell activation, Th2/Th17 cell involvement, and the recruitment of eosinophils and neutrophils, consistent with previous reports [24,32,33].

After confirming that neutrophils were involved in lung inflammation in our model, we investigated whether Nets were involved in this process. We first sought to determine whether Nets could be identified within lungs of NA. We detected positive immunofluorescence staining for markers of Nets including CitH3 and MPO in this NA mouse model (Fig 2f). Additionally, we detected whether Nets were adjacent to DCs in lungs of OVA/LPS-induced mice, which would indicate potential interactions. By staining citH3 and CD11c (a DC marker), we found Nets in close proximity to CD11c positive cells (Fig 2g).

We next evaluated whether the removal of Nets would inhibit lung inflammation of NA. We used Sivelestat, a neutrophil elastase inhibitor, which inhibited Net formation, as a systemic treatment [34]. Sivelestat administration alone did not significantly affect airway inflammation in normal mice (Fig 2a-f). Compared to the NA group, OVA/LPS mice treated with Sivelestat exhibited reduced Nets formation, accompanied by significantly reduced airway inflammation, neutrophil and eosinophil recruitment, and concentrations of IL-4, 6, 17, and 23 in BALF (Fig 2a-c, f). Additionally, sivelestat decreased the percentages of CD11c+MHCII+IL6-DCs, CD11c+ MHCII+IL23-DCs, Th2, and Th17 cells in lungs of OVA/LPS-induced mice (Fig 2d, e). These results suggested that Nets participate in the induction of pulmonary inflammatory milieu in NA. Pharmacologic inhibition of Nets with Silvelestat reduced DCs activation, Th17 differentiation and protected from neutrophilic airway inflammation in vivo.

### Nets-BMDCs potentiate Th17 differentiation by p38MAPK and NF-κB Signaling in the Context of OVA/LPS

**Fig 3:**
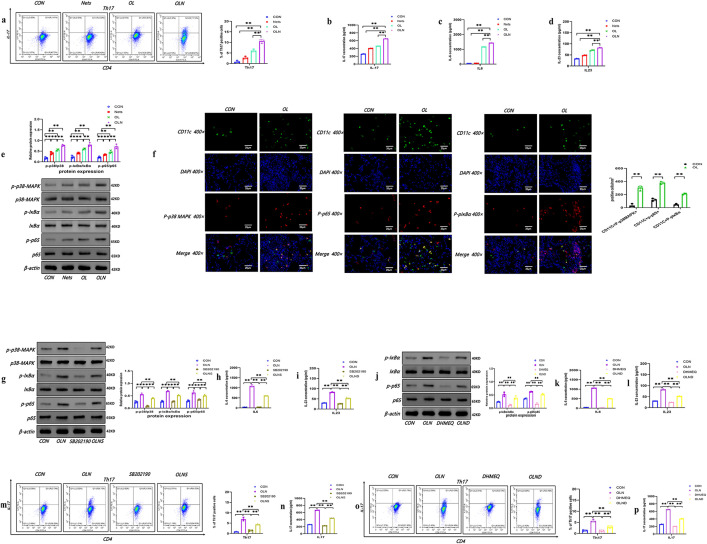
Nets-BMDCs in the context of OVA/LPS enhanced IL-6/23 secretions and promote Th17 differentiation by p38MAPK and NF-κB signal. BMDCs were pretreated with control culture, Nets, OVA/LPS, OVA/LPS/Nets and then coculture with naïve CD4 + T lymphocytes. a. Representative flow cytometric analysis and comparisons of Th17 in each group. b Comparisons of concentrations of IL-17 in each group. c. Comparisons of concentrations of IL-6 in each group, d. Comparisons of concentrations of IL-23 in each group. e. Representative Western blot images and comparisons of p-p38/p38 MAPK, p-IKBα/IKBα, p65/p65, β-actin in each group. f. The images of immunohistochemical staining and comparisons for P-p38 MAPK, P-pIKBα, P-p65 expression in the CD11c+ positive cells of CON and OVA/LPS induced lung, (P-p38 in the lung were identified with DAPI (blue), P-p38 (red) and CD11c+ (green) by confocal microscopy, P-p65 in the lung were identified with DAPI (blue), P-p65 (red) and CD11c+ (green) by confocal microscopy, P-p65 in the lung were identified with DAPI (blue), P-pIKBα (red) and CD11c+ (green) by confocal microscopy). OVA/LPS/Nets-stimulated BMDCs were pretreated with control culture, OVA/LPS/Nets, p38 inhibitor (SB202190), OVA/LPS/Nets/SB202190, and then coculture with naïve CD4 + T lymphocytes, g. Representative Western blot images and comparisons of p-p38/p38 MAPK, p-IKBα/IKBα, p65/p65, β-actin. in each group, h. Comparisons of concentrations of IL-6 in each group, i. Comparisons of concentrations of IL-23 in each group. OVA/LPS/Nets-stimulated BMDCs were pretreated with control culture, OVA/LPS/Nets, NF-κB inhibitor (DHMEQ), OVA/LPS/Nets/DHMEQ, and then coculture with naïve CD4 + T lymphocytes, j. Representative Western blot images and comparisons of p-IKBα/IKBα, p65/p65, β-actin in each group, k. Comparisons of concentrations of IL-6 in each group, l. Comparisons of concentrations of IL-23 in each group. m. Representative flow cytometric analysis and comparisons of Th17 in CON, OLN, SB202190, OLNS group. n. Comparisons of concentrations of IL-17 in in CON, OLN, SB202190, OLNS group. o. Representative flow cytometric analysis and comparisons of Th17 in in CON, OLN, DHMEQ, OLND group. p Comparisons of concentrations of IL-17 in each group. (Data were means ± SEM (n = 3); * * P < 0.01) (CON: control group, Nets: Neutrophil extracellular traps group, OL: OVA/LPS group, OLN:OVA/LPS/Nets group, SB202190:a p38-MAPK specific inhibitor group, DHMEQ: a NF-κB-specific inhibitor group, OLNS:OVA/LPS/Nets/SB202190 group, OLND:OVA/LPS/Nets/DHMEQ group).

Given that DC-triggered Th17 differentiation played a crucial role in NA pathogenesis, we investigated the impact of Nets on Th17 differentiation. Our results showed that BMDCs exposed to OVA/LPS/Nets increased Th17 percentages and IL-17 secretion compared to controls, Nets alone, and OVA/LPS alone (Fig 3a, b). Previous studies have found that IL-6 and 23 secreted by DCs induce Th17 differentiation, which was a major mechanism controlling the recruitment of neutrophils in asthma airways [35–37]. We next measured IL-6 and 23 levels in BMDCs separately stimulated with control culture, Nets, OVA/LPS, and OVA/LPS/Nets. BMDCs exposed to OVA/LPS/Nets exhibited significantly higher levels of IL-6 and IL-23 than those in all other conditions (Fig 3c, d).

Moreover, various interconnected signaling pathways, like the MAPK or NF-κB signaling pathway, directly or indirectly orchestrated the production of these cytokines by DCs in response to stimulation [38–41]. To elucidate the mechanism of OVA/LPS/Nets-stimulated BMDCs in IL-6 and IL-23 production, we assessed the activation of the p38MAPK and NF-κB pathway. Treatment with OVA/LPS/Nets markedly activated p-p38-MAPK, p-p65, and p-IκBα compared to controls, Nets alone, and OVA/LPS alone (Fig 3e). Importantly, the activation of p38-MAPK, p-p65, and p-IκBα, was also observed in the CD11c+positive cells in lung tissue of NA (Fig 3f). Additionally, pretreatment of OVA/LPS/Nets-stimulated BMDCs with p38 inhibitor (SB202190) or NF-κB inhibitor (DHMEQ) significantly inhibited IL-6 and 23 productions, accompanied by decreased activity of p38-MAPK, p-p65, and p-IκBα without affecting total protein levels (Fig 3g-l). Moreover, inhibitors of the p38MAPK or NF-κB pathway also significantly reduced the percentage of Th17 cells induced by OVA/LPS/Nets-stimulated BMDCs (Fig 3m-p). These outcomes supported that Nets-DC-Th17 axis potentiated DCs activation and the induction of IL-17 inflammation in NA.

### Effect of XQLT on Airway Inflammation in NA Mouse Models

**Fig 4:**
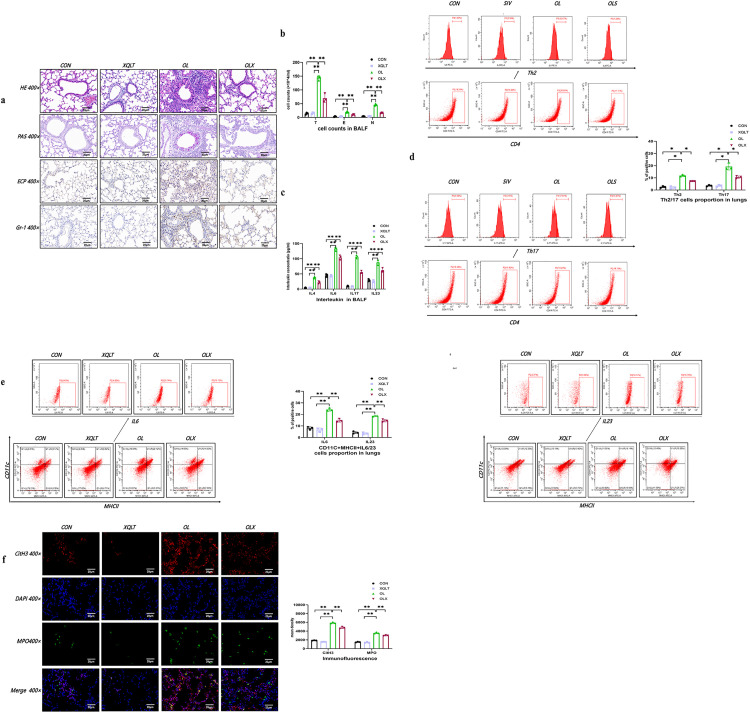
Xiao Qing Long Tang (XQLT) reduced airway inflammation, Nets formation, DC activation and Th differentiation in OVA/LPS-induced neutrophilic asthma models. a. Histological images of lung sections stained with haematoxylin and eosin (H&E), periodic acid–Schiff (PAS), immunohistochemistry for ECP and Gr-1 expression. b. Comparisons of total cell counts (T), eosinophil counts (E), and neutrophil counts (N) (10^4^ cells/ml) in BALF across each group. c. Comparisons of concentrations of IL-4, IL-17, IL-6, and IL-23 in each group. d. Representative flow cytometric analysis and comparisons of Th2/17 cells in lung tissues of each group. e. Representative flow cytometric analysis and comparisons of CD11c+MHC-II + IL-6/23 DCs in in lung tissues of each group. f. Identification of Nets in lung tissues co-stained with DAPI (blue), cit-Histone3 (red), and MPO (green) using confocal microscopy. (Data are means ± SEM (n = 3); * *P < 0.01) (CON: control group, XQLT: Xiao Qing Long Tang group, OL: OVA/LPS group, OLX: OVA/LPS/XQLT group).

To evaluate the preliminary anti – inflammatory effect of XQLT, we administered XQLT to NA mouse models, and the results showed that it reduced airway inflammation (Fig 4a, b). Cytokine analysis in BALF revealed that XQLT treatment reduced levels of IL-4, 6, 17 and 23, as well as a decline in Th2 and Th17 cell proportions in lung of OVA/LPS group (Fig 4c, d). XQLT also decreased the proportion of IL-6 and IL-23-producing cells in DCs compared to NA models (Fig 4e). Importantly, immunofluorescence analysis found that compared with NA group, XQLT group showed a reduction in the expression of Nets in the lungs of mice (Fig 4f). These findings highlighted XQLT’s potential to alleviate Th17 related airway inflammation by modulating Nets-DCs-Th17 axis.

### XQLT Inhibited Nets-DCs-Th17 Axis and p38MAPK/NF-κB Signaling In Vitro

**Fig 5:**
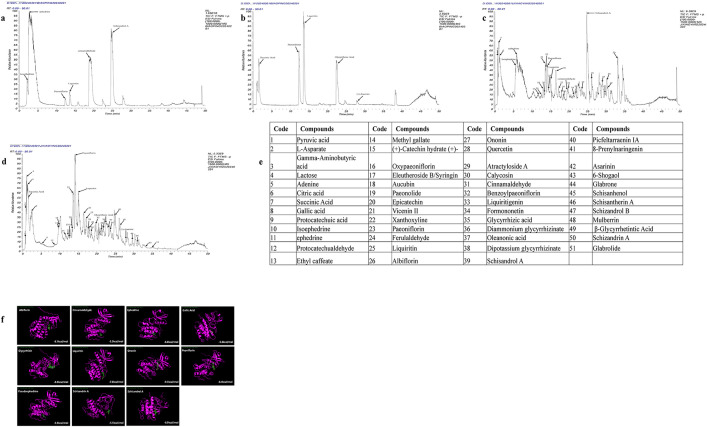
The chemical composition analysis of XQLT by UPLC-MS and Molecular docking of partial components in XQLT with MAPK14. a. Chromatogram of standard products in positive ion mode. b. Chromatogram of standard products in negative ion mode. c. Chromatogram of XQLT in positive ion mode. d. Chromatogram of XQLT in negative ion mode. e. A comprehensive list of chemical components presented in XQLT along with their respective encodings. f. Molecular docking of Albiflorin, Cinnamaldehyde, Ephedrine, Gallic Acid, Glycyrrhizin, Liquiritin, Ononin, Peoniflorin, Pseudoephedrine, Schisandrin A, Schisandrol A in XQLT with MAPK14(The text in the upper left corner of the image represents the components, and the number in the lower right corner represents the combined free energy).

**Fig 6:**
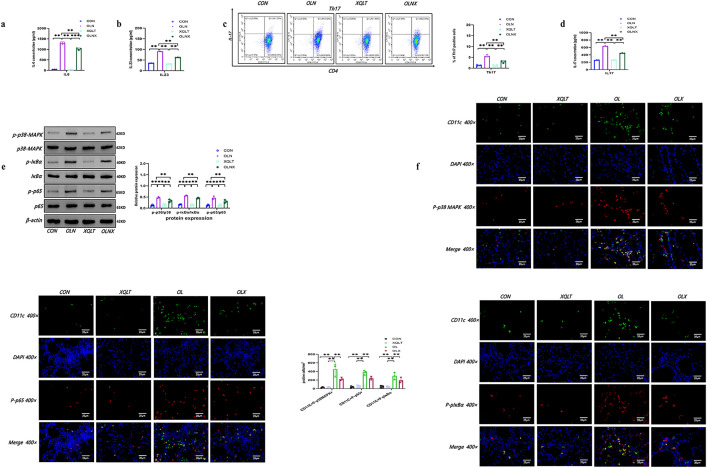
XQLT inhibited Nets-DCs-Th17 axis in BMDCs and p38MAPK/NF-κB signal in the CD11c+ positive cells of lung. BMDCs were pretreated with control culture (CON), OVA/LPS/Nets (OLN), XQLT, OVA/LPS/Nets/XQLT(OLNX) and then coculture with naïve CD4 + T lymphocytes. a. comparisons of concentrations of IL-6 in each group, b. comparisons of concentrations of IL-23 in each group, c. Representative flow cytometric analysis and comparisons of Th17 cells in each group, d. Comparisons of concentrations of IL-17 in each group, f. Representative Western blot images and comparisons of p-p38/p38 MAPK, p-IKBα/IKBα, p-p65/p65, β-actin in each group, f. The images of immunohistochemical staining and comparisons for P-p38 MAPK, P-pIKBα, P-p65 expression in the CD11c+ positive cells of lung, (P-p38 in the lung were identified with DAPI (blue), P-p38 (red) and CD11c+ (green) by confocal microscopy, P-p65 in the lung were identified with DAPI (blue), P-p65 (red) and CD11c+ (green) by confocal microscopy, P-p65 in the lung were identified with DAPI (blue), P-pIKBα (red) and CD11c+ (green) by confocal microscopy) (Data are means ± SEM (n = 3); ** P < 0.01) (CON: control group, XQLT: Xiao Qing Long Tang group, OLN: OVA/LPS/Nets group, OLNX:OVA/LPS/Nets/XQLT group).

To experimentally elucidate the direct effect of XQLT on Net-DCs-Th17 Axis, we first analyzed its composition using UPLC-MS. A total of 51 compounds were identified in XQLT based on their mass fragmentation patterns. The total ion chromatograms under both positive and negative ionization modes, along with the mass spectrum of XQLT, were presented in Fig 5a-e. Supplementary Material S3 Table provides detailed MS spectral data and retention times for the bioactive compounds identified in XQLT. Our previous network pharmacology study identified the MAPK signaling pathway as a target of XQLT, which was closely linked to inflammatory responses [42]. To explore the interaction between specific compounds within XQLT and the MAPK pathway, we conducted molecular docking analysis involving 11 pairs: Albiflorin, Cinnamaldehyde, Ephedrine, Gallic Acid, Glycyrrhizin, Liquiritin, Ononin, Peoniflorin, Pseudoephedrine, Schisandrin A, and Schisandrol A with MAPK14 (human, UniProt P49137). The binding energies from these receptor-ligand interactions were depicted in Fig.5f. All of them were below −5.0 kcal/mol, indicating stable binding interactions.

we further investigated its effects on OVA/LPS/Nets-stimulated BMDCs-CD4 + T cells cocultured model. XQLT reduced cytokines milieu of IL-6 and 23 in OVA/LPS/Nets-stimulated BMDCs (Fig 6a, b). Moreover, XQLT treatment significantly suppressed the elevated IL-17 levels and the proportion of Th17 cells induced by OVA/LPS/Nets-stimulated BMDCs (Fig 6c, d). Additionally, XQLT inhibited the activation of p38-MAPK, p-p65, and p-IκBα, while the total protein levels remained unchanged compared to the OVA/LPS/Nets-stimulated BMDCs group (Fig 6e). This trend was also observed in the CD11c+positive cells in lung tissue of NA models treated with XQLT. Treatment with XQLT markedly reduced the activation of p38-MAPK, p-p65 and p-IKBa in CD11c+positive cells of lung tissue compared to OVA-LPS group (Fig 6f). These outcomes indicated that XQLT inhibited Nets-DCs-Th17 Axis possibly through p38MAPK and NF-κB signaling.

## Discussion

NA is an inflammatory airway disease characterized by the heterogeneous infiltration of neutrophils and eosinophils. It involves DC activation through antigen presentation to naïve CD4 + T cells in response to various inhaled stimuli [24]. The release of Nets by neutrophils prompts its development and glucocorticoid resistance [17], which indicates that intervention on Nets-related pathway can be potential targets for alternative therapies. Here, we showed that the inhibition of Nets effectively reduced neutrophil-dominated airway inflammation, DC activation, and Th17 differentiation by utilizing mice and cell models. Additionally, treatment with XQLT in our study alleviated inflammation effects of Nets-DC-Th17 axis in NA.

Previous studies found that Nets aggravated inflammatory lung injury by stimulating immune cells and regulating inflammatory cytokines. For example, Nets could facilitate lymphocyte aggregation [43] and induce Th1 and Th17 differentiation, contributing to COPD initiation [44]. In patients with type 1 diabetes, Nets enhanced DCs maturation and inflammatory cytokine production, thereby promoting a proinflammatory state and IFNγ-producing T lymphocytes [45]. Nets have also been linked to enhanced secretion of IL-1 and 8 in human bronchial epithelial cells, correlating with the severity of acute respiratory distress syndrome [29]. In our study, we first evaluated the effect of Nets on airway inflammation in NA by using Sivelestat to reduce Nets generation. We found that the reduction of Nets was accompanied by a decrease in airway neutrophil inflammation, DCs activation, and Th17 differentiation in NA model. Importantly, Nets and DCs are in close proximity in the lungs of OVA/LPS – treated mice. These results suggested that Nets had an interaction with DCs activation.

Advances in understanding innate and adaptive immunity have demonstrated that DCs increased inflammatory cytokine production and played a central role in immune tolerance or imbalance. Nets downregulated LPS-induced DCs maturation, diminishing their ability to stimulate CD4 + T lymphocyte proliferation and reducing Th1 and Th17 polarization while promoting Th2 polarization [46]. Conversely, Nets induced by free fatty acids stimulated DCs activation, promoting Th1/17 polarization in acute lung injury models [47]. In type 1 diabetes patients, Nets-induced DC activation leaded to Th1 polarization, but this effect was absent in healthy individuals [45]. Similarly, in collagen-induced arthritis, Nets exposure promoted DC maturation and effector Th1 differentiation rather than Th17 differentiation [30]. Based on these findings, the influence of Nets on DCs activation and effector Th cells differentiation varied depending on the immune microenvironment. Our results showed that DCs exposed to Nets in an OVA/LPS context significantly increased secretion of IL-6 and IL-23, along with elevated Th17 levels. In immune regulation, IL-6 drived Th17 cell development, crucial in chronic inflammation, while IL-23 promoted memory Th17 cell proliferation [48,49]. Notably, IL-6 and IL-23 also contributed to corticosteroid resistance in asthma models characterized by neutrophil infiltration [50,51]. Together, these findings supported the notion that in asthma, DCs responded to Nets, augmenting its activation and Th17 cell differentiation.

Intrigued by the proinflammatory response of DCs to Nets in the OVA/LPS context, we investigated its immunoregulatory mechanisms to identify therapeutic targets for mitigating inflammation in NA. Mechanistically, the activation of the p38-MAPK or NF-κB signal played a central role in DC immune modulation, increasing levels of proinflammatory cytokines IL-6 and IL-23 in response to environmental signals [38,52–55]. Typically, NF-κB binded to IκBα, preventing its nuclear translocation; however, phosphorylation of IκBα leaded to its degradation, facilitating NF-κB nuclear translocation. Inflammatory stimuli further enhanced p38 phosphorylation, amplifying NF-κB-mediated transactivation and promoting expression of target genes such as IL-6 and IL-23 [56]. Our study revealed that OVA/LPS/Nets synergistically affected p38 MAPK and NF-κB pathway phosphorylation. Additionally, inhibitors of this pathway blocked the augmentation of IL-6/IL-23 and Th17 differentiation induced by OVA/LPS/ Nets. These findings suggested a role for p38-MAPK and NF-κB signal in the anti-inflammatory mechanism within the body. Collectively, Nets-DCs-Th17 axis played a potentially crucial role in contributing to inflammation of NA.

Currently, severe NA cases exhibited poor responsiveness to standard therapies. TCM has attracted attention as an adjunctive therapy to disrupt the inflammatory cycle in asthma. Previous studies have shown that XQLT could reduce eosinophilic infiltration, prevent airway structural changes, and modulate Th2-driven inflammation in eosinophilic asthma [57]. However, no research has explored the protective effects of XQLT on NA. In our study, XQLT alleviated histopathological changes, reduced Nets formation, cytokine secretion, and suppressed Th17 differentiation in NA models. Cell experiments have found that XQLT regulated Nets-DCs-Th17 axis to exert protective effects. To elucidate the signaling mechanisms underlying XQLT’s anti-inflammatory effects in OVA/LPS/Nets-stimulated DCs, we performed UPLC-MS analysis to identify its chemical composition and validated the protective effect of XQLT in OVA/LPS/Nets stimulated DCs. Here, Molecular docking and WB verifications consistently demonstrated that XQLT inhibits the p38MAPK/NF-κB pathway in vitro and vivo by preventing p38MAPK and NF-κB phosphorylation in DCs stimulated with OVA/LPS/Nets. These findings suggested that XQLT maintained cytokine homeostasis and breaks the inflammatory cycle in NA.

Despite these promising findings, our study had several limitations. DCs interacted with naïve T cells through pathways involving MHC antigen presentation, inflammatory signal transduction, and cytokine production. While our study focused on cytokine/chemokine profiles, the role of co-stimulatory molecules in DC-T cell interactions required further investigation. Furthermore, activated DCs released cytokines, that mediated the differentiation of CD4 + T cells into different subgroups. Our article only explored the effect of XQLT on Th17 cell differentiation, lacking evidence to demonstrate its impact on DC-Th1, Th2, or Treg interactions. Sivelestat sodium had pleiotropic effects. Besides reducing the formation of Nets, it also reduced airway inflammation by inhibiting neutrophil elastase [34,58]. Therefore, in our animal model, the effects of sivelestat could not be simply attributed to its inhibition of Nets. Additionally, during flow cytometry analysis, the cell viability status and the presence of debris could interfere with cell discrimination and increases background noise, affecting result accuracy. Finally, the therapeutic effects of XQLT targeted different pathways through the combined actions of multiple components. In our study, we highlighted the pivotal role of the p38MAPK-NF-κB pathway in OVA/LPS/Nets-stimulated DCs, demonstrating its effect on demonstrating its effect on promoting IL-6 and IL-23 secretion to enhance Th17 differentiation. However, the potential impact of XQLT on other pathways remained a topic for further investigation.

In conclusion, our findings suggested that XQLT exerted therapeutic effects by inhibiting Nets-DC-Th17 axis in NA. More research was needed to combine the multi-target effects of traditional Chinese medicine prescriptions with the clinical phenotypes of diseases, which will be beneficial for discovering new therapeutic targets.

## Supporting information

S1 Table The ingredient composition of XQLT. (PDF)

S2 Table Sample liquid chromatography-mass spectrometry method of XQLT. (DOCX)

S3 Table The chemical components of XQLT. (PDF)

S1 File. Raw image (PDF)

## Abbreviations

ANOVA: One‐way analysis of variance
BALF: Bronchoalveolar lavage fluid
BMDCs: Bone marrow-derived dendritic cells
CitH3: citrullinated histone H3
DAB: 3,3 Diaminobenzidine
DAPI: 4,6-diamidino-2-phenylindole
DCs: Dendritic cells
dsDNA: Double-strand DNA
E: Eosinophils
EDTA: Ethylene Diamine Tetraacetic Acid
ELISA: Enzyme-linked immunosorbent assay
FBS: Fetal bovine serum
GM-CSF: Granulocyte-macrophage colony-stimulating factor
H&E: Haematoxylin and eosin
IL: Interleukin
LPS: Lipopolysaccharide
MAPK: Mitogen-activated protein kinase
MPO: Myeloperoxidase
N: Neutrophils
NA: Neutrophilic asthma
Nets: Neutrophil extracellular traps
NF-κB: Nuclear factor-κB
OVA: Ovalbumin
PBS: Phosphate buffer saline
PAS: Periodic acid–Schiff
p-p38: Phospho-p38 MAPK
p-p65: Phospho-p65
p-IkBa: Phosphor-p-IkBa
PMA: Phorbol 12-myristate13-acetate
PVDF: Polyvinylidene fluoride
SD: Standard deviation
SDS-PAGE: Sodium dodecyl sulfatepolyacrylamide electrophoresis
TBC: Total cell count
TCM: Traditional Chinese Medicine
Th: T helper
UPLC-MS: Ultraperformance liquid chromatography and mass spectrometry
XQLT: Xiao-qing-long-tang
