# Supplementary material for: Xiao Qing Long Tang ameliorates neutrophil extracellular trap-dendritic cells-T helper 17 cell axis in Neutrophilic Asthma
Source: PLoS One. 2025 Nov 6;20(11):e0336333. doi: 10.1371/journal.pone.0336333 (PMC12591476; doi:10.1371/journal.pone.0336333)
Supplement: S1 Table — (PDF) [file pone.0336333.s001.pdf]

The ingredient Composition of XQLT

herbal names	botanical plant names	weight(g)
Baishao	Paeonia lactiflora Pall	6
Fa banxia	Pinellia ternata (Thunb.) Makino	9
Ganjiang	Zingiber officinale Roscoe	6
Guizhi	Neolitsea cassia (L.) Kosterm	6
Mahuang	Ephedra sinica Stapf	6
Wuweizi	Schisandra Chinensis (Turcz.) Baill	3
Xixin	Asarum heterotropoides F.Schmidt	3
Zhi gancao	Glycyrrhiza uralensis Fisch	6
