# Supplementary material for: Xiao Qing Long Tang ameliorates neutrophil extracellular trap-dendritic cells-T helper 17 cell axis in Neutrophilic Asthma
Source: PLoS One. 2025 Nov 6;20(11):e0336333. doi: 10.1371/journal.pone.0336333 (PMC12591476; doi:10.1371/journal.pone.0336333)
Supplement: S2 Table — (DOCX) [file pone.0336333.s002.docx]

**Sample liquid chromatography-mass spectrometry method of XQLT**

| **Project** | **Description** | **Manufacturer** |
| --- | --- | --- |
| Liquid phase system | Dionex Ultimate 3000 RSLC (HPG) | Thermo Fisher Scientific |
| Mass spectrometry system | Thermo Scientific Q Exactive Focus | Thermo Fisher Scientific |
| Ion source | HESI-II | Thermo Fisher Scientific |

**chromatographic column**

| **Type** | **Specifications** | **Manufacturer** |
| --- | --- | --- |
| Waters Acquity uplc hss T3 1.8um | 100*2.1 mm | waters |

**Ion source parameter settings**

| Parameter | Value |
| --- | --- |
| Spray Voltage | 3.0 kV (+)/2.5 kV (-) |
| Capillary Temperature | 320°C |
| Sheath Gas | 35 arb |
| AUX Gas | 10 arb |
| Sweep Gas | 0 arb |
| Probe Heater Temperature | 350°C |
| S-Lens | 60 |

**Gradient elution table**

| Time (min) | Flow (mL/min) | C（Organic phase）acetonitrile (0.1% formic acid) | B（aqueous phase）0.1% formic acid water |
| --- | --- | --- | --- |
| 0 | 0.3 | 5 | 95 |
| 2 | 0.3 | 5 | 95 |
| 42 | 0.3 | 95 | 5 |
| 47 | 0.3 | 95 | 5 |
| 47.1 | 0.3 | 5 | 95 |
| 50 | 0.3 | 5 | 95 |
| Column temperature：40°C | | | |

**Gradient elution table**

| Mass spectrometry scanning parameters | Value |
| --- | --- |
| Scan mode | Full MS-ddms^2^ |
| Full MS scan range | 100 to 1500*m*/*z* |
| Spectrum data type | Profile |
| Resolution | Full MS: 70,000 |
|  | MS/MS: 17,500 |
| AGC target | Full MS:1e^6^ |
|  | MS/MS:2e^5^ |
| Maximum IT | Full MS: 100 ms |
|  | MS/MS: 50 ms |
| Loop count | 3 |
| MSX count | 1 |
| Isolation width | 1.5*m*/*z* |
| NCE (Stepped NCE) | 20, 40, 60 |
| Minimum AGC target | 8e^3^ |
| Intensity Threshold | 1.6e^5^ |
| Dynamic exclution | 5 s |
