# Supplementary material for: Xiao Qing Long Tang ameliorates neutrophil extracellular trap-dendritic cells-T helper 17 cell axis in Neutrophilic Asthma
Source: PLoS One. 2025 Nov 6;20(11):e0336333. doi: 10.1371/journal.pone.0336333 (PMC12591476; doi:10.1371/journal.pone.0336333)
Supplement: S3 Table — (PDF) [file pone.0336333.s003.pdf]

The chemical components of XQLT

Number	Compounds	RT (min)	Molecular Weight(g/mol)	[M-H]+	MS^2	Formula	Area
1	Pyruvic acid	0.408	148.03609	（M-H）-	147.0447 102.9475 89.0232 61.9870	C3 H4 O3	51521.64024
2	L-Asparate	0.832	133.03642	（M-H）-	132.0209 115.0024 88.0390 71.0125	C4 H7 N O4	1540510.59
3	Gamma-Aminobutyric acid	0.886	121.07422	（M-H）-	104.1074 87.0445 69.0341 60.0816	C4 H9 N O2	1799233.597
4	Lactose	1.102	388.1215	（M-H）-	341.1085 179.0552 119.0337 101.0231 89.0230 71.0125 59.0125	C12 H22 O11	21543925.49
5	Adenine	1.173	118.028	（M+NH4）+	136.0618 119.0354 92.0246 67.0300	C5 H5 N5	38959771.01
6	Citric acid	1.386	192.02625	（M-H）-	191.0109 173.0081 111.0075 87.0074 85.0281 57.0332	C6 H8 O7	1181571775
7	Succinic Acid	1.63	118.0266	（M-H）-	117.0181 99.0075 73.0282	C4H6O4	156170864
8	Gallic acid	2.505	170.02067	（M-H）-	169.0133 125.0232 108.0205 95.0124 79.0174 69.0332	C7 H6 O5	822203648.9
9	Protocatechuic acid	5.382	154.0256	（M-H）-	153.0182 123.0432 109.0282 91.0176 81.0332	C7 H6 O4	32525714.48
10	Isoephedrine	5.53	165.1154	（M+H）+	56.0503	C10H15NO	1267266176
11	ephedrine	5.86	165.1154	（M+H）+	56.0503	C10H15NO	1770976896
12	Protocatechualdehyde	8.717	138.03058	（M-H）-	137.0232 119.0127 109.0282 93.0332 81.0331 65.0382	C7 H6 O3	65594436.83
13	Ethyl caffeate	9.123	208.07298	（M-H）-	207.0294 163.0391 118.9918 73.0282	C11 H12 O4	11991.07265
14	Methyl gallate	9.268	184.03631	（M-H）-	183.0289 168.0054 139.0389 124.0153	C8 H8 O5	3035321.556
15	(+)-Catechin hydrate (+)-	11.581	290.07892	（M-H）-	289.0715 245.0813 203.0705 151.0389 123.0439 109.0282	C15 H14 O6	58388747.14
16	Oxypaeoniflorin	12.259	496.15798	（M-H）-	495.1504 137.0232 93.0332	C23 H28 O12	48463717.16
17	Eleutheroside B/Syringin	12.543	418.14726	（M-H）-	417.1073 209.0814 194.0580 179.034 161.0234 71.0123 59.0124	C17 H24 O9	7393138.75
18	Aucubin	12.617	392.12971	（M-H）-	345.1548 323.1344 101.0230 89.0230 71.0124 59.0124	C15 H22 O9	821748.1431
19	Paeonolide	13.301	441.11342	（M+ACN+H）+	483.1476 317.0839 189.0523 73.0290	C20 H28 O12	4991653.617
20	Epicatechin	13.323	290.07893	（M-H）-	289.0715 245.0814 203.0705 151.0389 123.0438 109.0281	C15 H14 O6	15912526.24
21	Vicenin II	13.618	594.1575	（M-H）-	593.1505 473.1083 383.0767 353.0662 279.0765	C27 H30 O15	28459323.17
22	Xanthoxyline	13.78	196.07341	（M+H）+	197.0806 151.0752 133.0647 105.0701	C10 H12 O4	12727203.51
23	Paeoniflorin	14.157	480.16317	（M+NH4）+	179.0699 151.0751 133.0646 105.0701 85.0289 79.0548	C23 H28 O11	823735732.8
24	Ferulaldehyde	14.693	178.06284	（M+H）+	179.0702 151.0752 133.0647 105.0702 79.0548	C10 H10 O3	12582413.56
25	Liquiritin	15.042	418.12609	（M-H）-	417.1188 255.0658 135.0075 119.0489 91.0175	C21 H22 O9	376146894.3
26	Albiflorin	16.472	480.1631	（M+H）+	197.0806 179.0697 133.0646 105.0337	C23 H28 O11	757544709.2
27	Ononin	17.226	430.12654	（M+H）+	269.0804 254.0566 213.0906 118.0413	C22 H22 O9	174435194.3
28	Quercetin	17.79	286.08395	（M-H）-	301.0348 255.0293 211.0394 139.0025	C15 H10 O7	5760408.532
29	Atractyloside A	17.962	494.23604	（M-H）-	447.228 315.1808 161.0443 101.0230 71.0124	C21 H36 O10	9320773.138
30	Calycosin	18.485	284.06828	（M+H）+	285.0752 270.0517 253.0491 225.0541 137.0230	C16 H12 O5	27566621.2
31	Cinnamaldehyde	19.42	132.0575	（M+H）+	133.0647 115.0544 105.0701 103.0545 91.0546 79.0548 55.0187	C9H8O	947492288
32	Benzoylpaeoniflorin	19.585	584.18934	（M+NH4）+	267.0860 249.0754 179.0700 105.0337	C30 H32 O12	136749326.9
33	Liquiritigenin	21.296	256.07321	（M-H）-	255.0657 153.0181 135.0074 119.0489 91.0175	C15 H12 O4	146037521.3
34	Formononetin	21.971	268.07338	（M+H）+	269.0803 237.0539 226.0619 118.0413	C16 H12 O4	23489141.35
35	Glycyrrhizic acid	22.55	822.40394	（M-H）-	71.0124	C42 H62 O16	193819763.4
36	Diammonium glycyrrhizinate	23.03	822.40394	（M-H）-	821.3956 351.0568 289.0555 193.0344 175.0237 113.0231 85.0281	C42 H62 O16	89984946.2
37	Oleanonic acid	23.383	454.34471	（M+H）+	456.2052 191.1793 173.1330 145.1019 107.0862 95.0863	C30 H46 O3	1456325.659
38	Dipotassium glycyrrhizinate	23.788	822.40394	（M-H）-	821.3955 351.0564 193.0343 113.0231 85.0281	C42 H60 O16	140536312.6
39	Schisandrol A	24.96	432.2148	（M+H）+	415.2104 384.1925 369.1690 257.0811	C24H32O7	816821120
40	Picfeltarraenin IA	25.139	808.42366	（M-H）-	807.4161 351.0563 193.0344 113.0231 85.0281	C41 H62 O13	35515662.39
41	8-Prenylnaringenin	26.315	340.13082	（M-H）-	339.1232 187.1119 151.0025 132.0568 107.0125 65.0019	C20 H20 O5	11713129.57
42	Asarinin	27.95	354.1103	（M-H）-	353.1026 338.0785 269.0451 241.0501	C20H18O6	55476996
43	6-Shogaol	29.081	276.17259	（M+H）+	137.0596 122.0363 94.0417	C17 H24 O3	46002650.28
44	Glabrone	29.449	336.09962	（M-H）-	335.0923 319.0606 305.0453	C20 H16 O5	5678264.82
45	Schisanhenol	29.603	402.20439	（M+H）+	403.2108 371.1851 333.1329 302.1142 287.0910 227.0697	C23 H30 O6	120968473.4
46	Schisantherin A	30.009	536.2049	（M+NH4）+	415.1746 371.1485 340.1301 325.1076 105.0342	C30 H32 O9	125443326.3
47	Schizandrol B	30.497	514.22054	（M+NH4）+	415.1744 371.1486 340.1301 325.1063	C28 H34 O9	111219717.8
48	Mulberrin	31.11	422.17067	（M-H）-	421.1651 227.0706 193.0861 149.0960 125.0960	C25 H26 O6	11511001.1
49	β-Glycyrrhetintic Acid	32.842	470.33969	（M+H）+	471.3480 317.2110 149.0951 135.1167 121.1013 107.0858 95.0860	C30 H46 O4	29831575.95
50	Schizandrin A	33.209	416.21995	（M+H）+	417.2266 402.2035 347.1487 316.1301 301.1068 285.1115 242.0930	C24 H32 O6	993409237.9
51	Glabrolide	34.854	468.32332	（M-H）-	467.3160 423.3263 407.2950 353.2470	C30 H44 O4	1907417.826
