# Supplementary material for: Xiao Qing Long Tang ameliorates neutrophil extracellular trap-dendritic cells-T helper 17 cell axis in Neutrophilic Asthma
Source: PLoS One. 2025 Nov 6;20(11):e0336333. doi: 10.1371/journal.pone.0336333 (PMC12591476; doi:10.1371/journal.pone.0336333)
Supplement: S1 File. Raw image — (PDF) [file pone.0336333.s004.pdf]

1. original image of WB

Figure3e

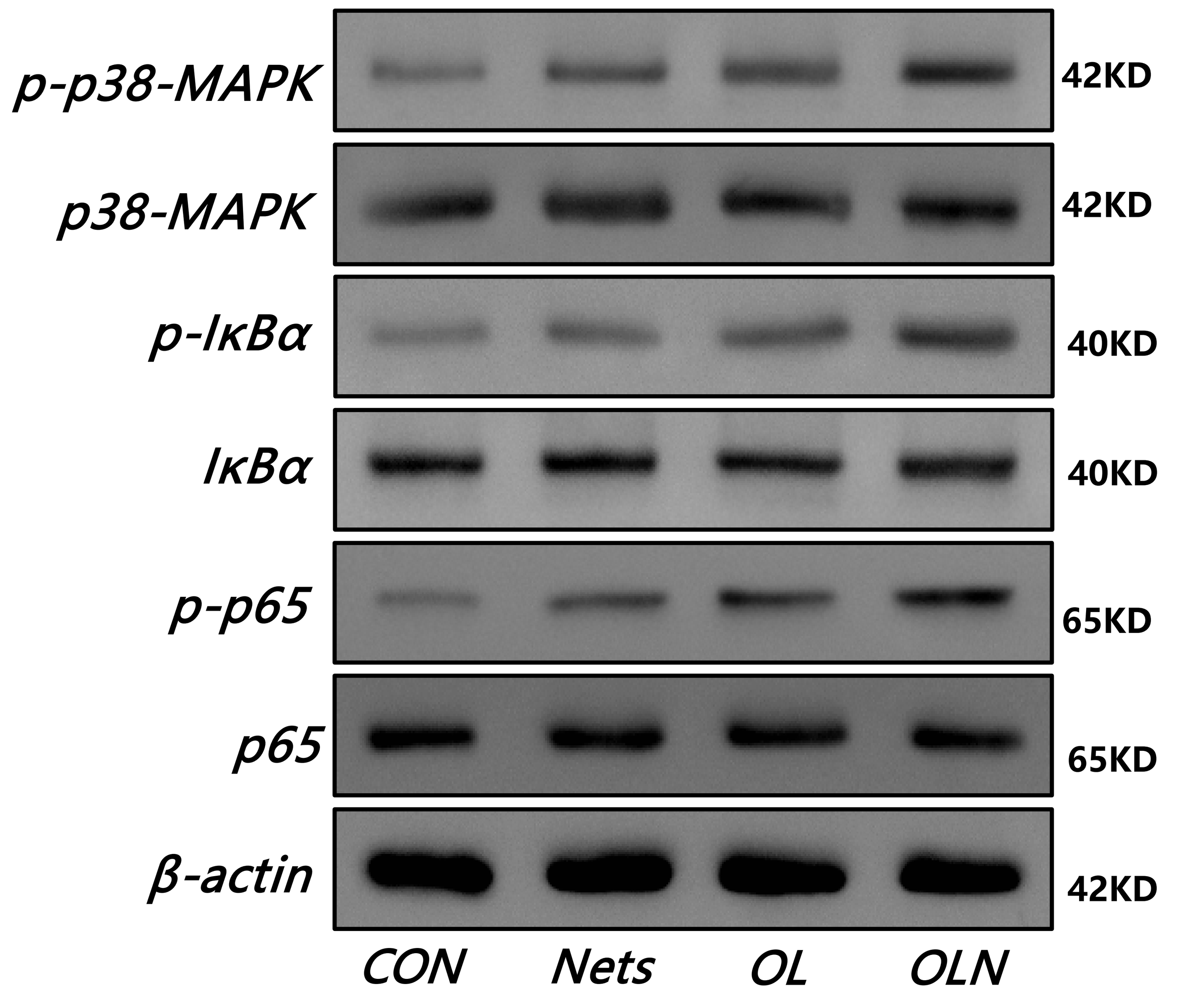

Figure3e1

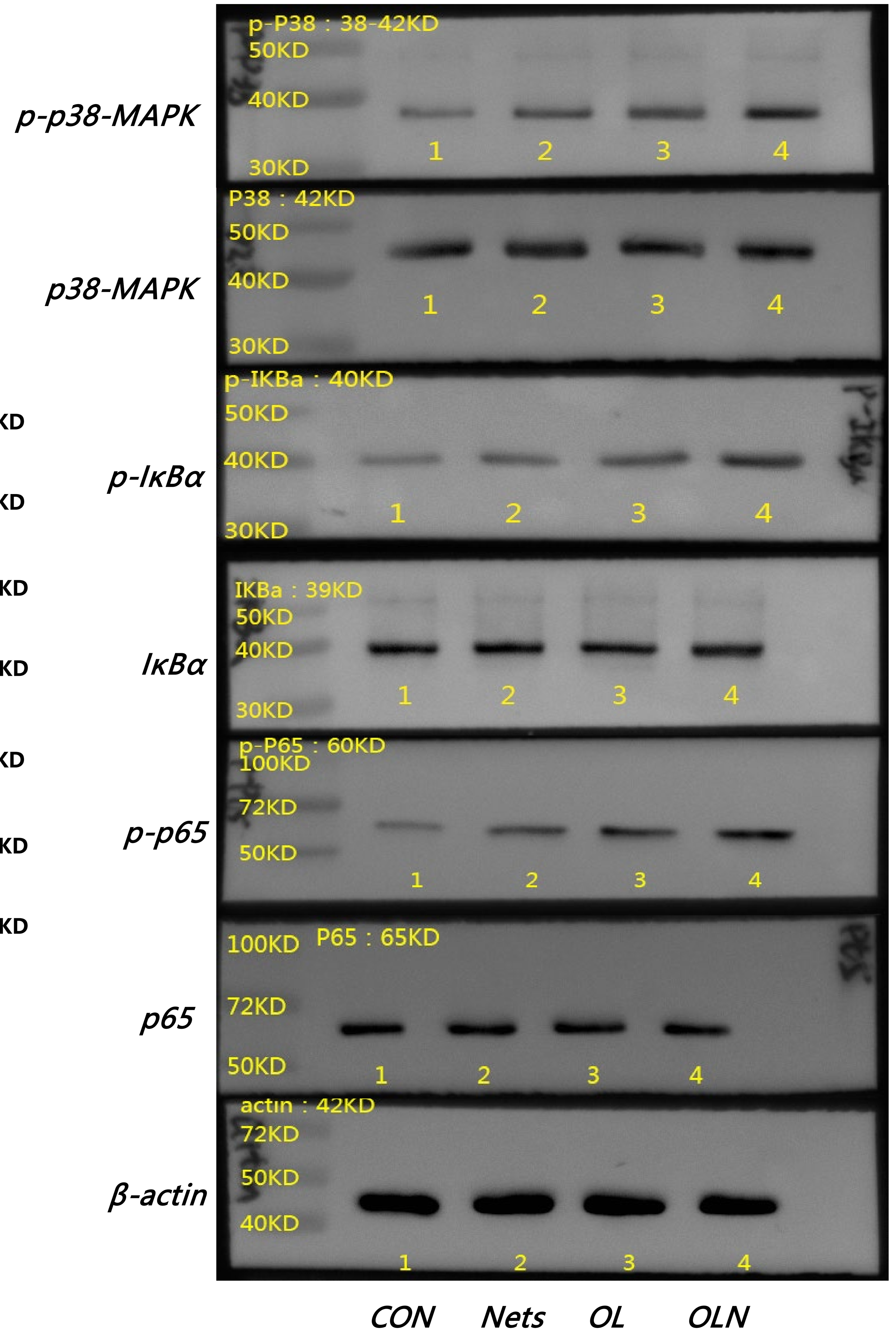

Figure3e2

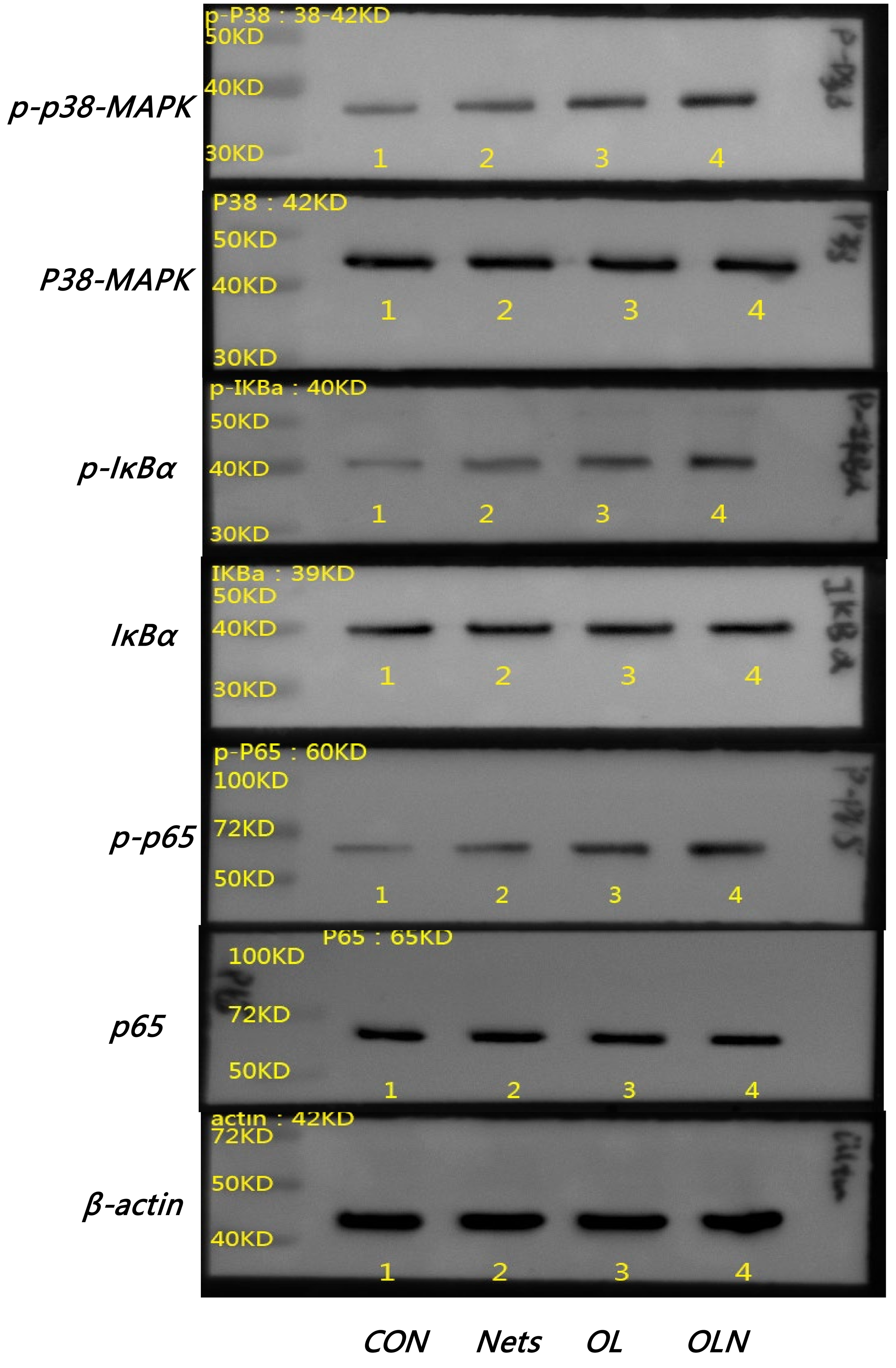

Figure3e3

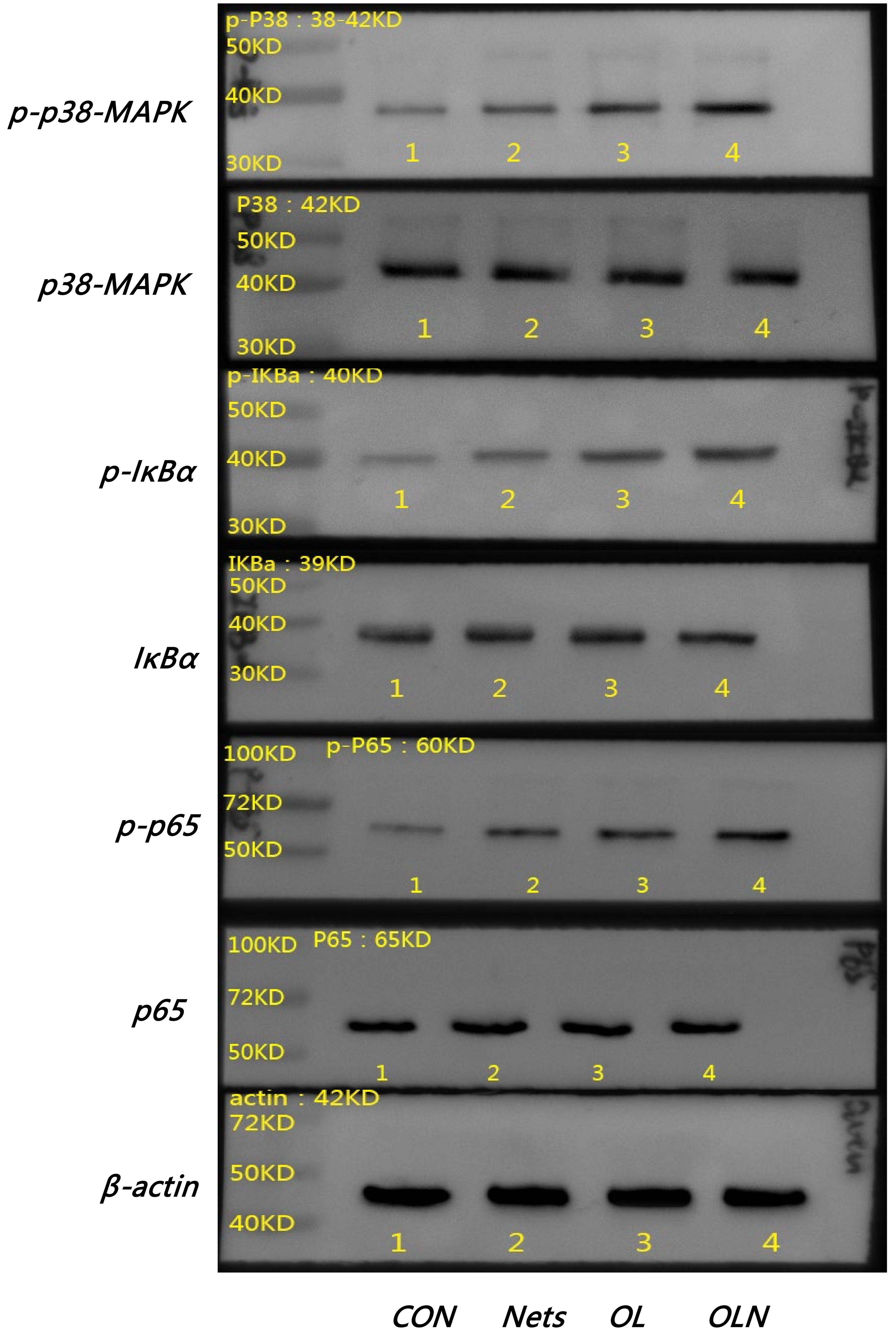

Figure3g

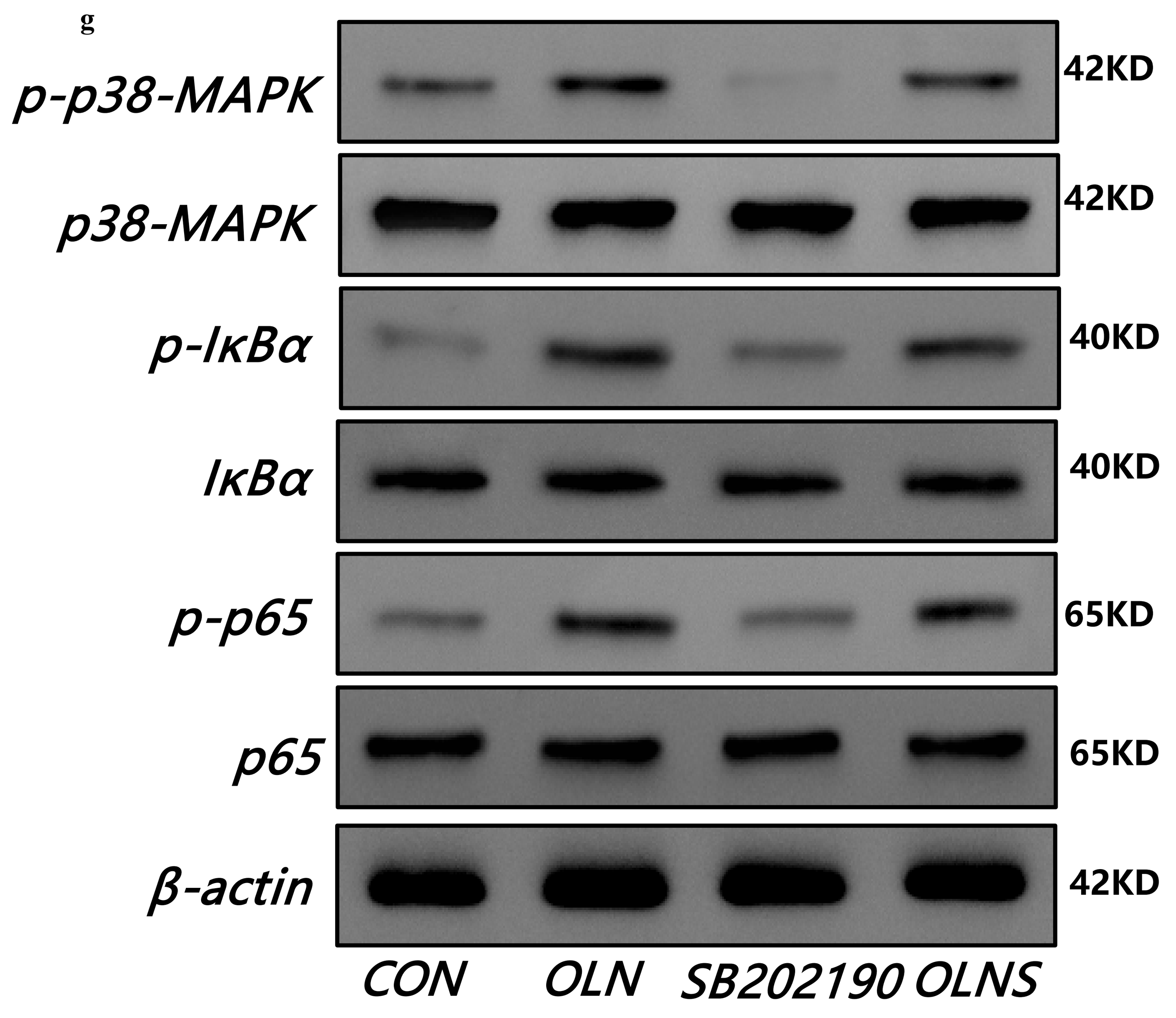

Figure3g1

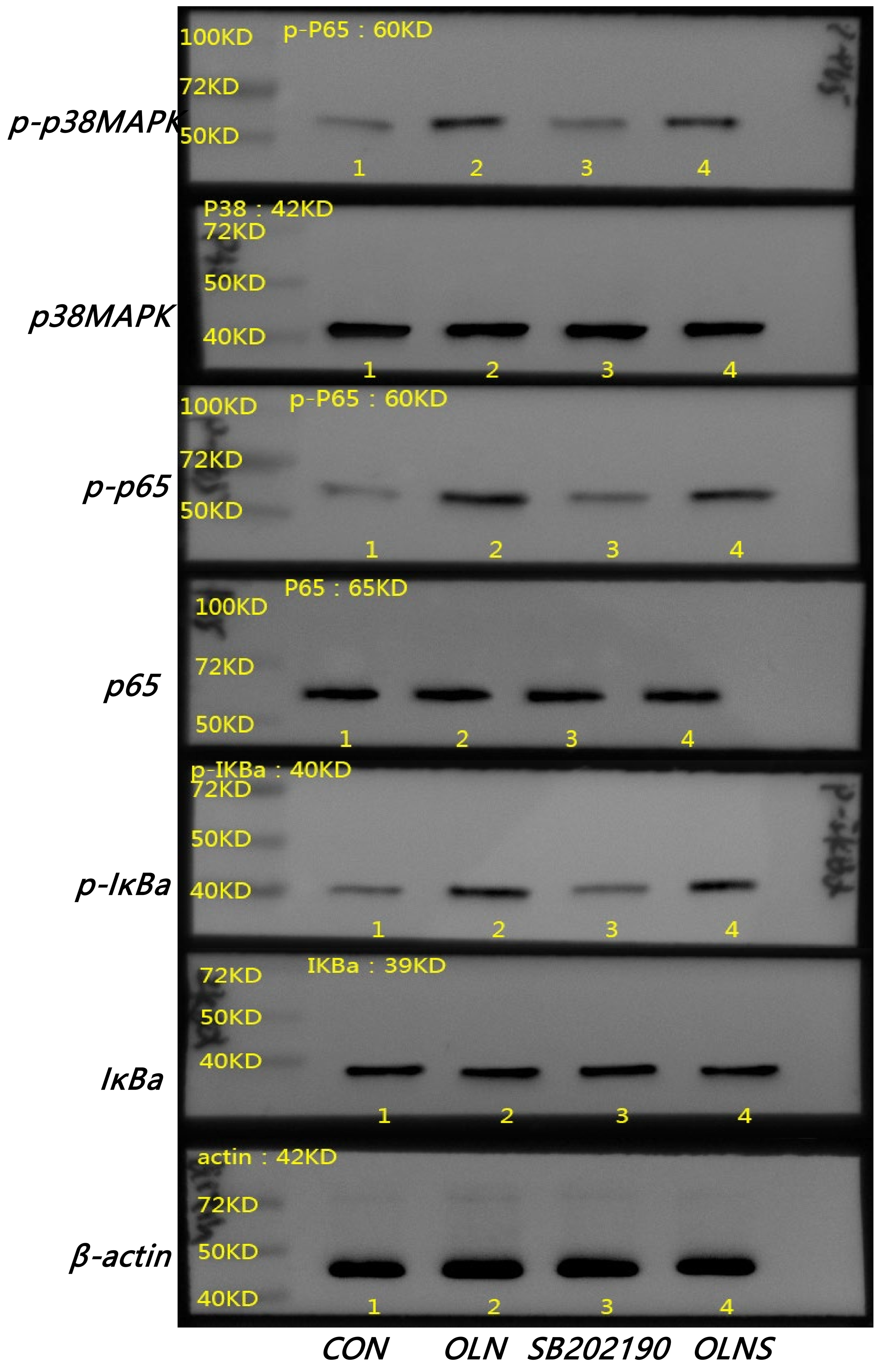

Figure3g2

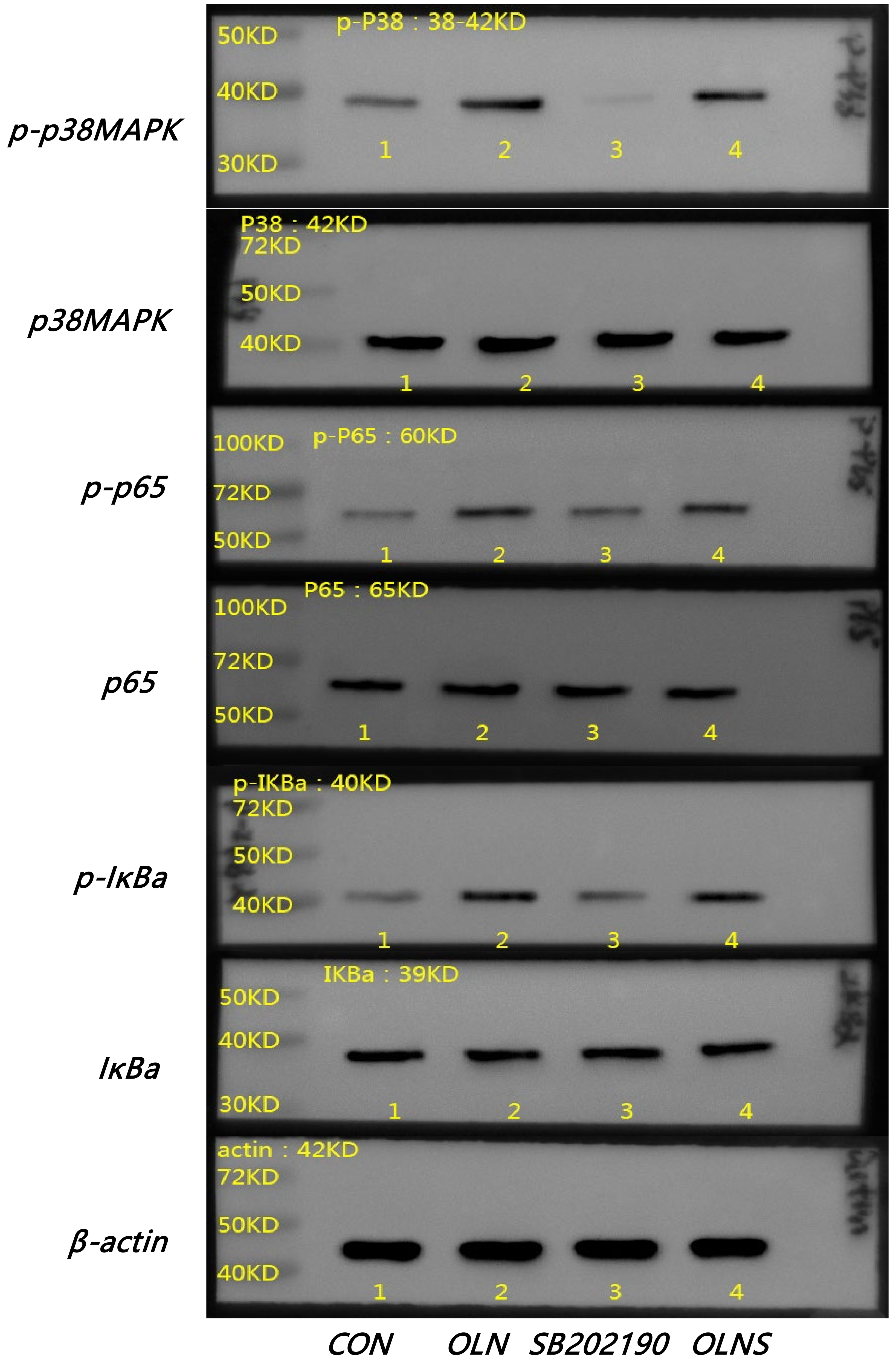

Figure3g3

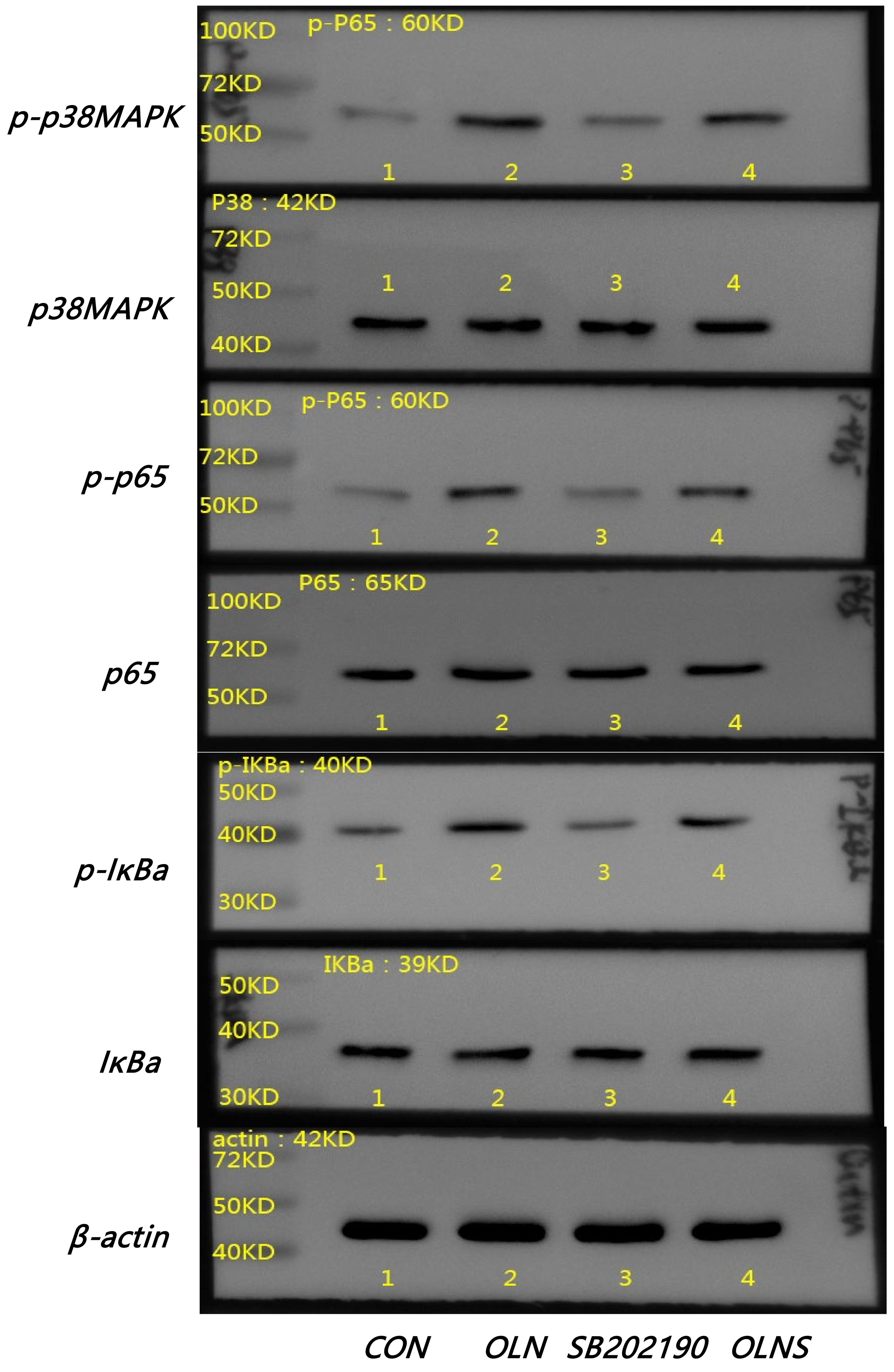

Figure3j

j

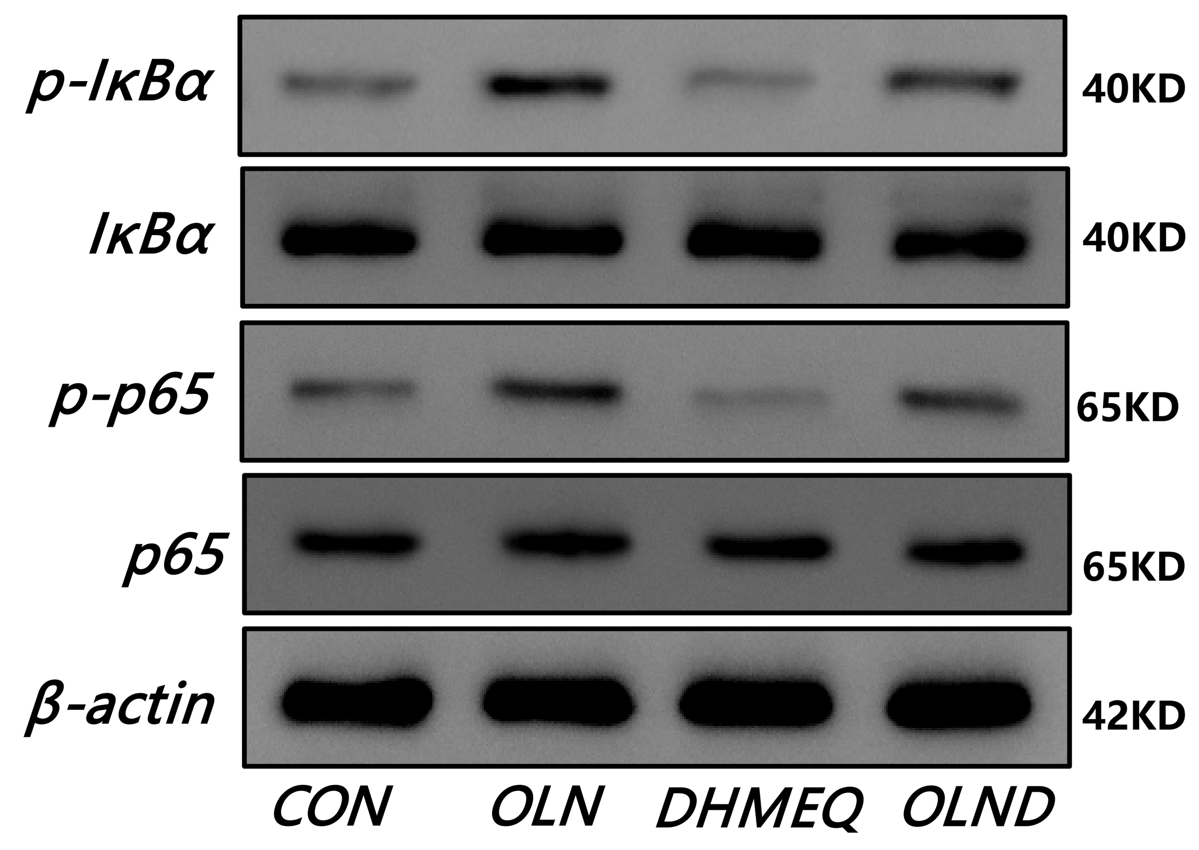

Figure3j1

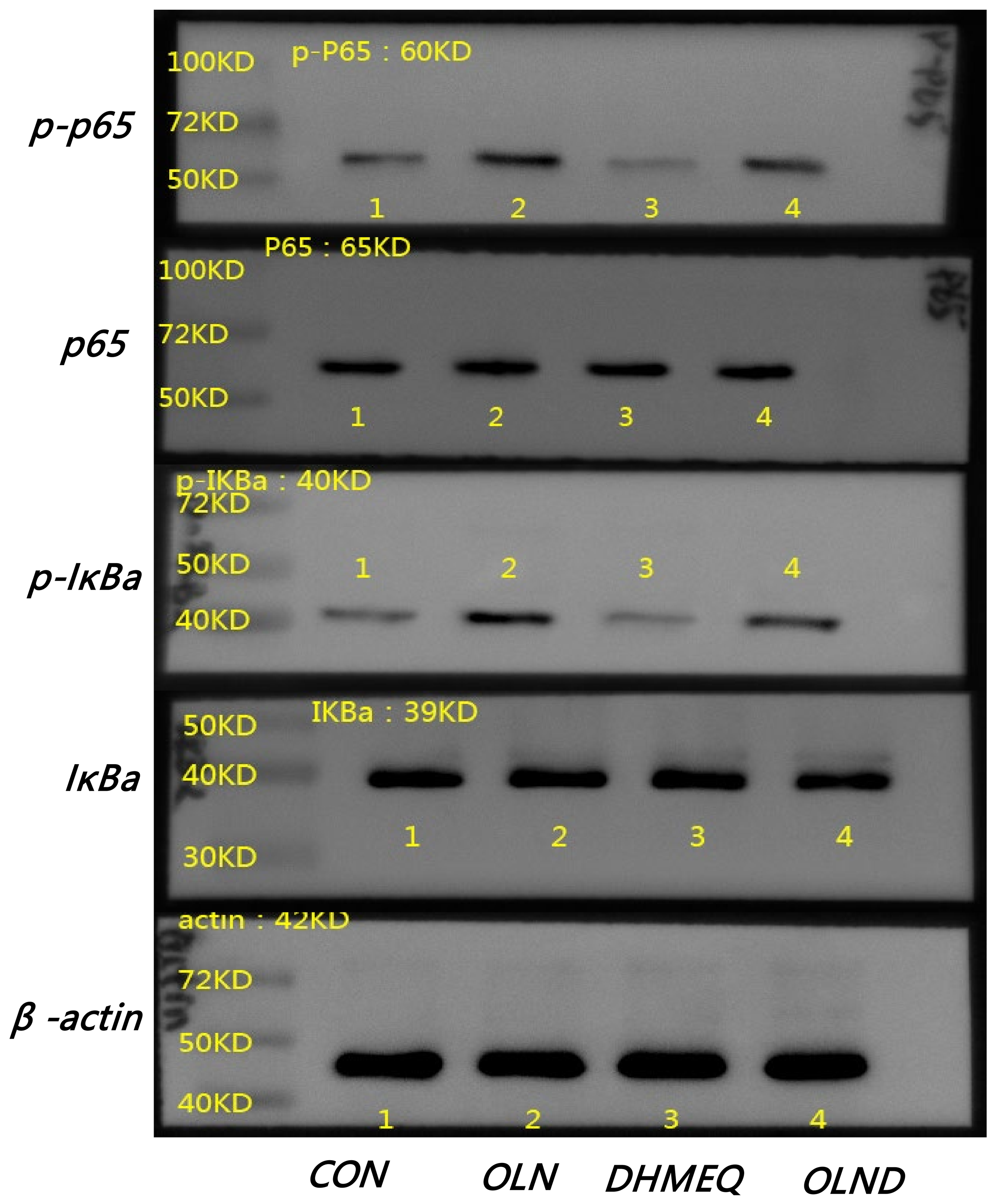

Figure3j2

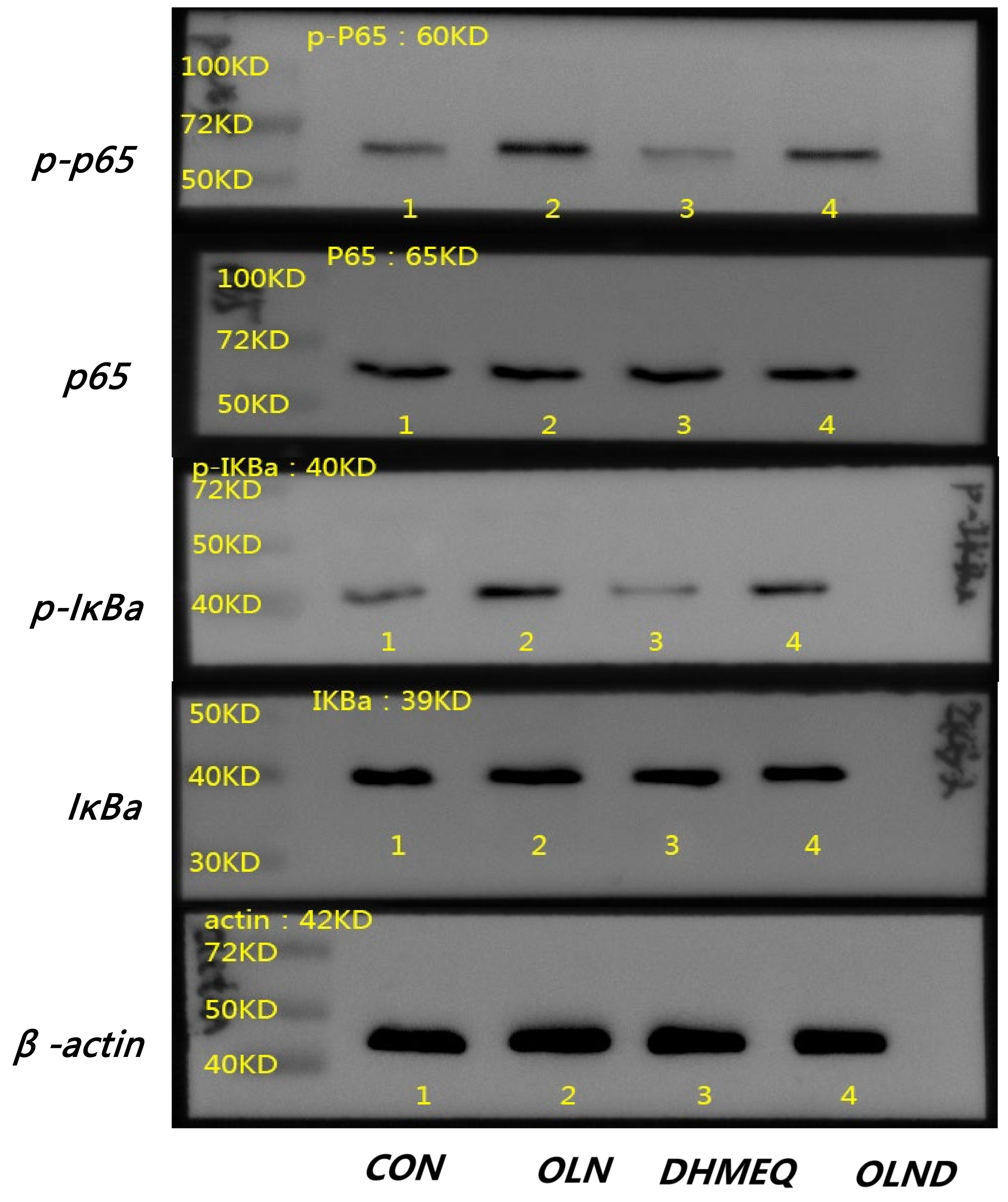

Figure3j3

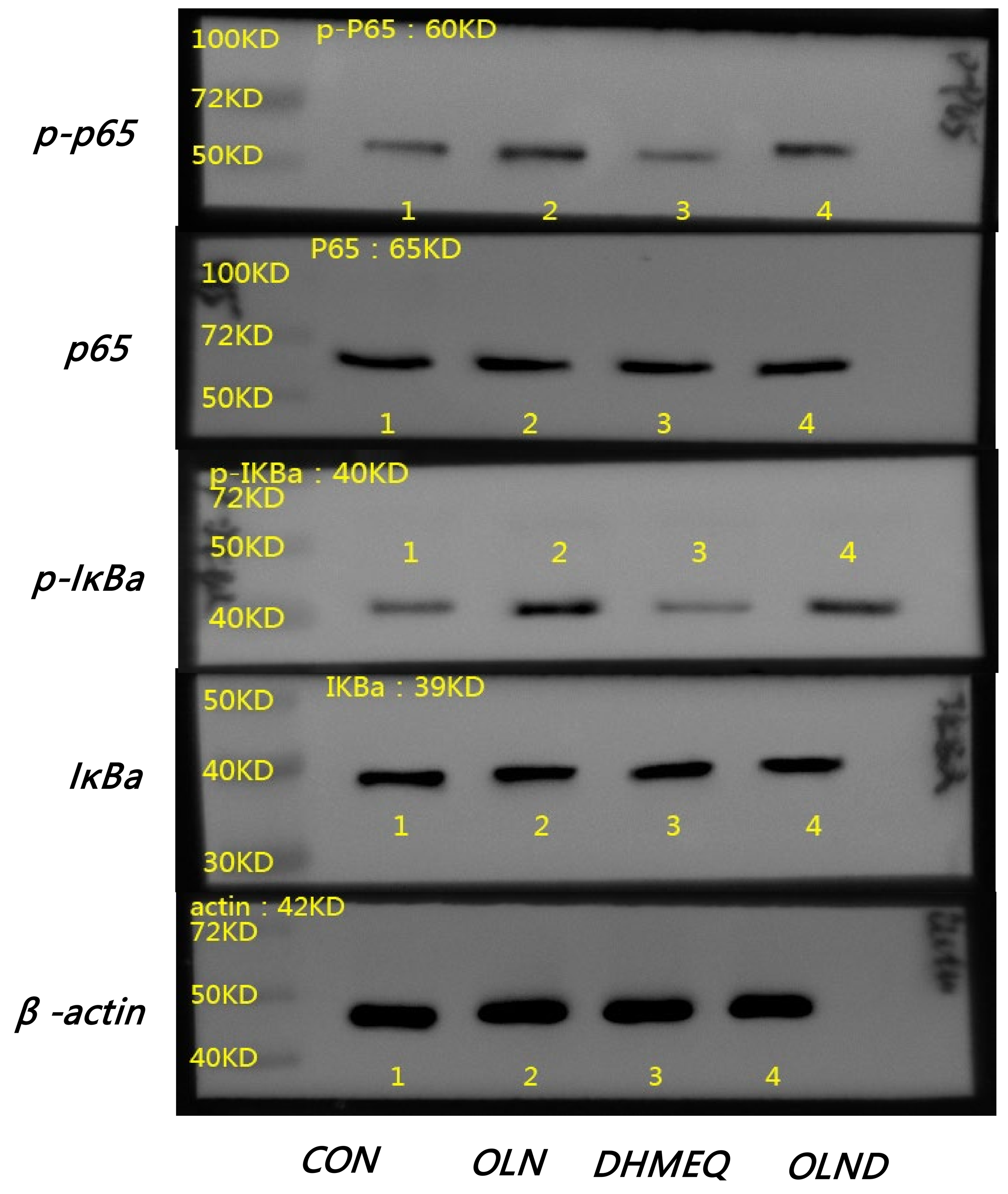

Figure6e

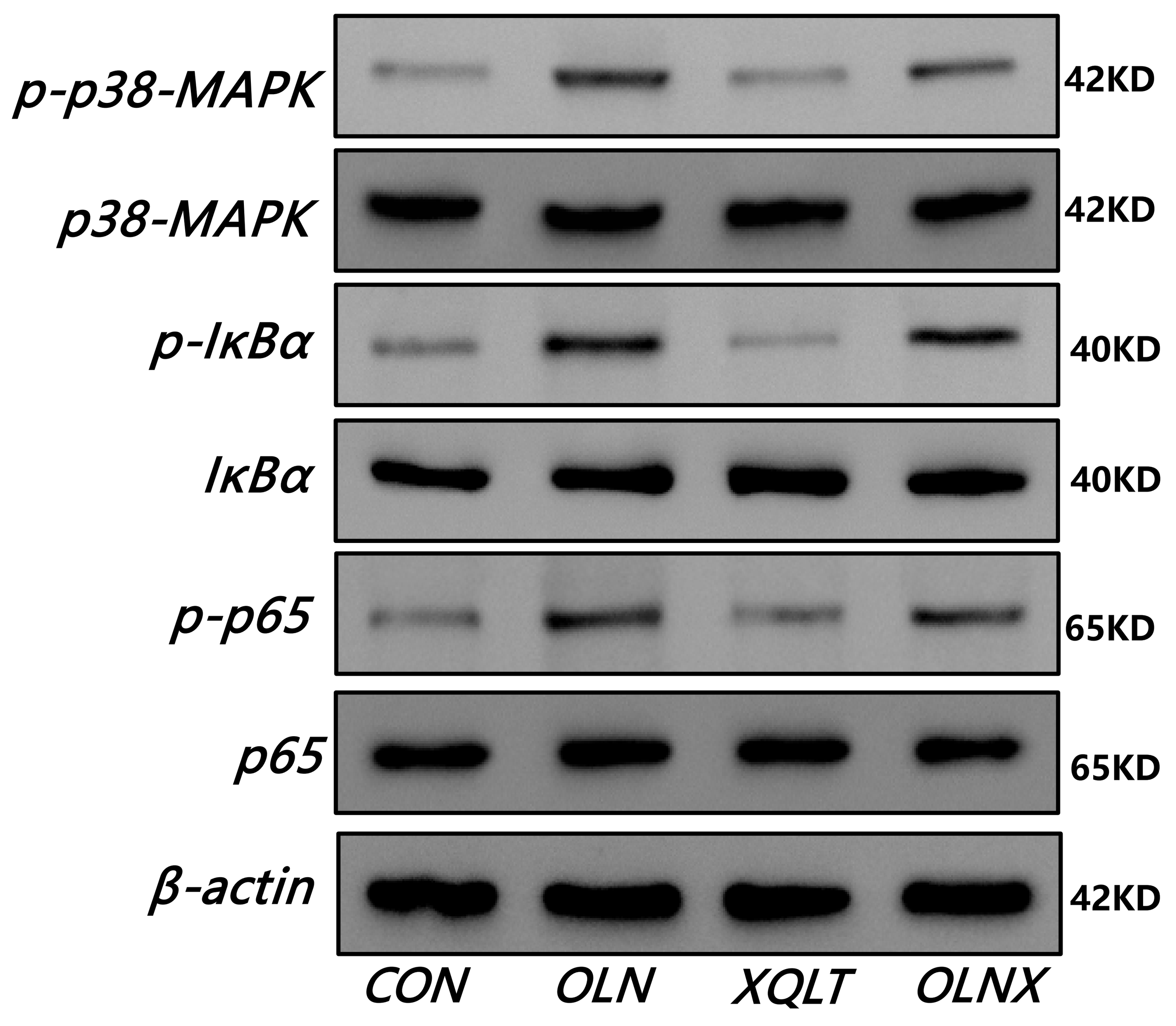

Figure6e1

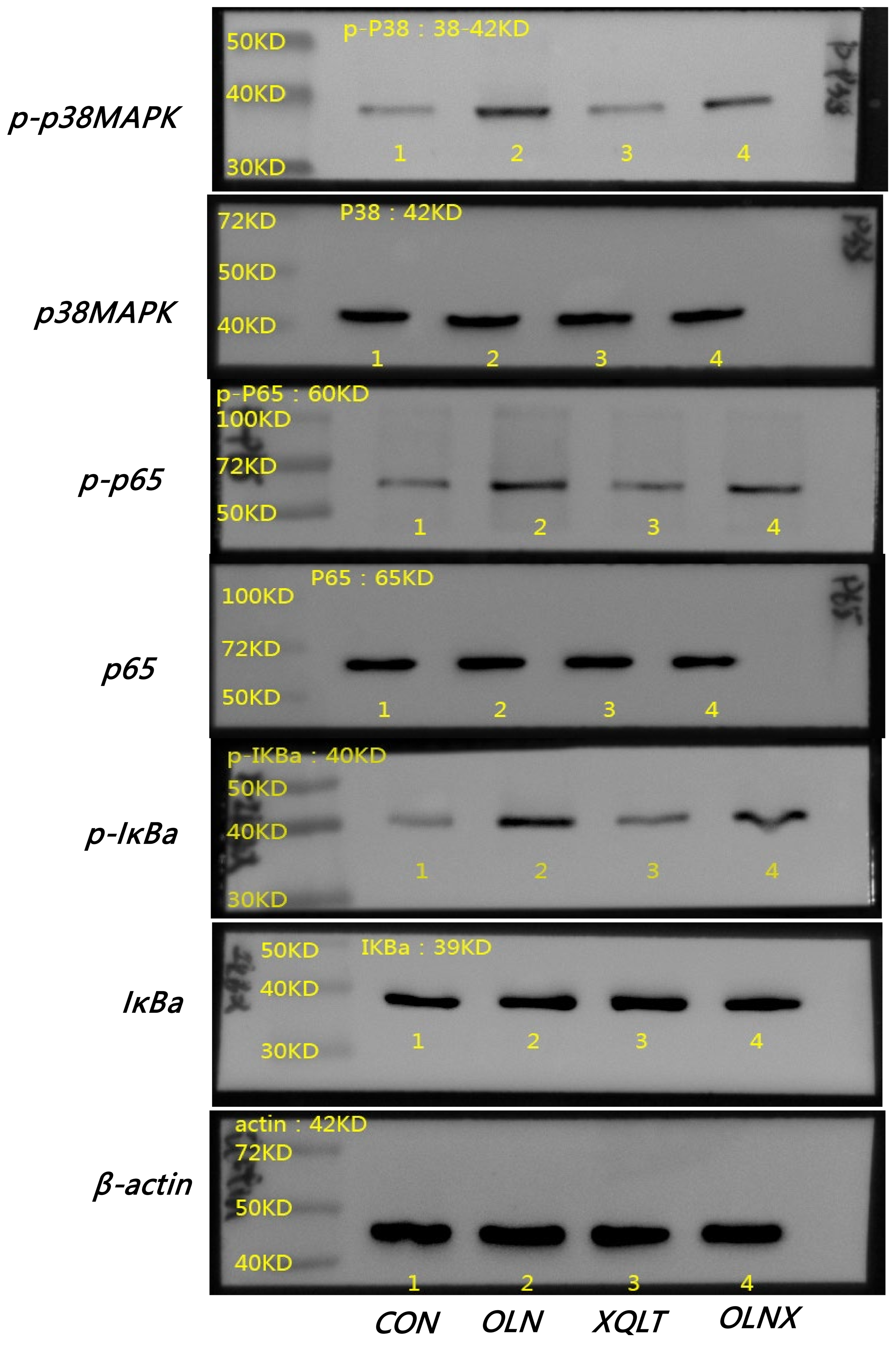

Figure6e2

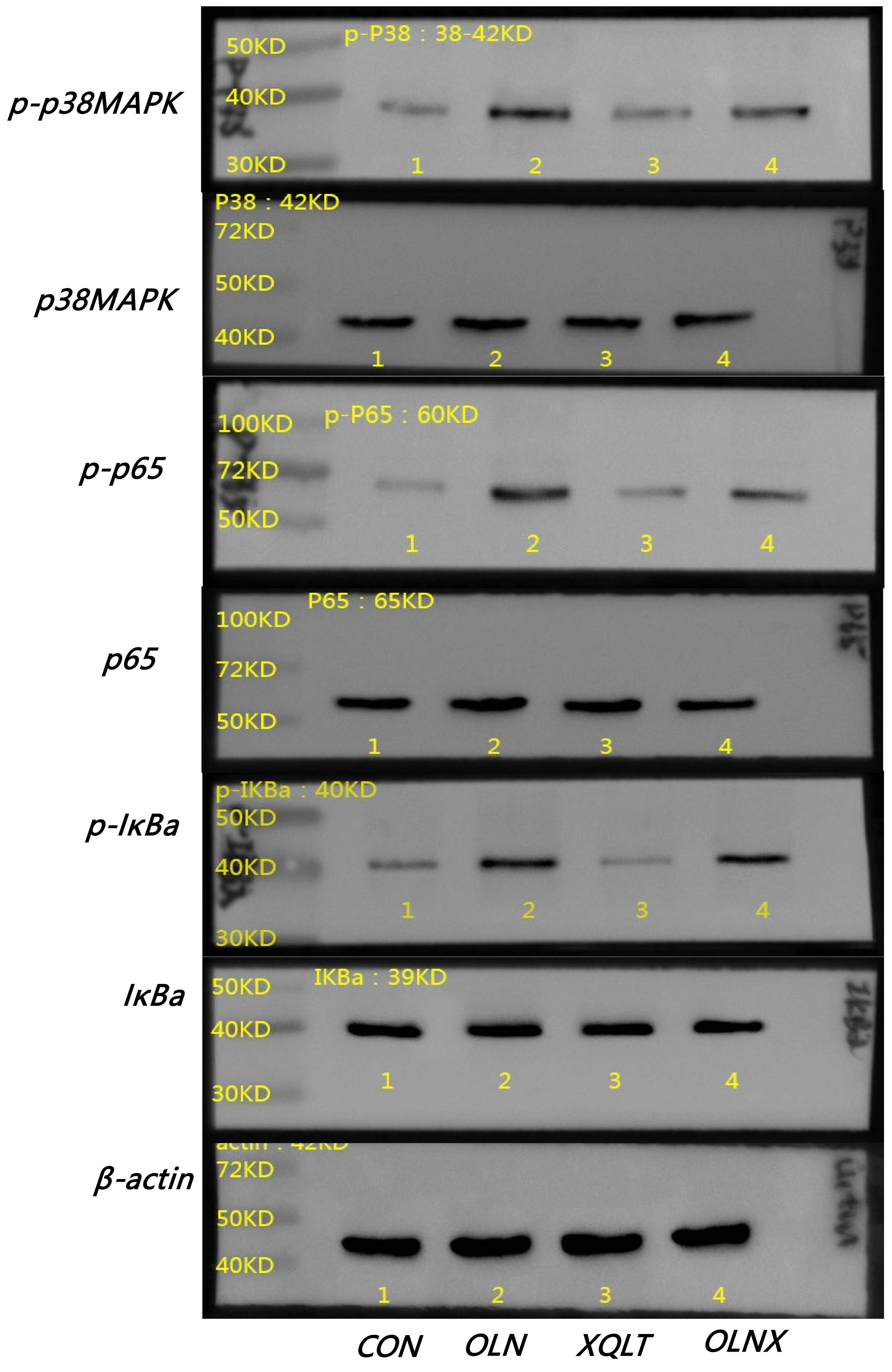

Figure6e3

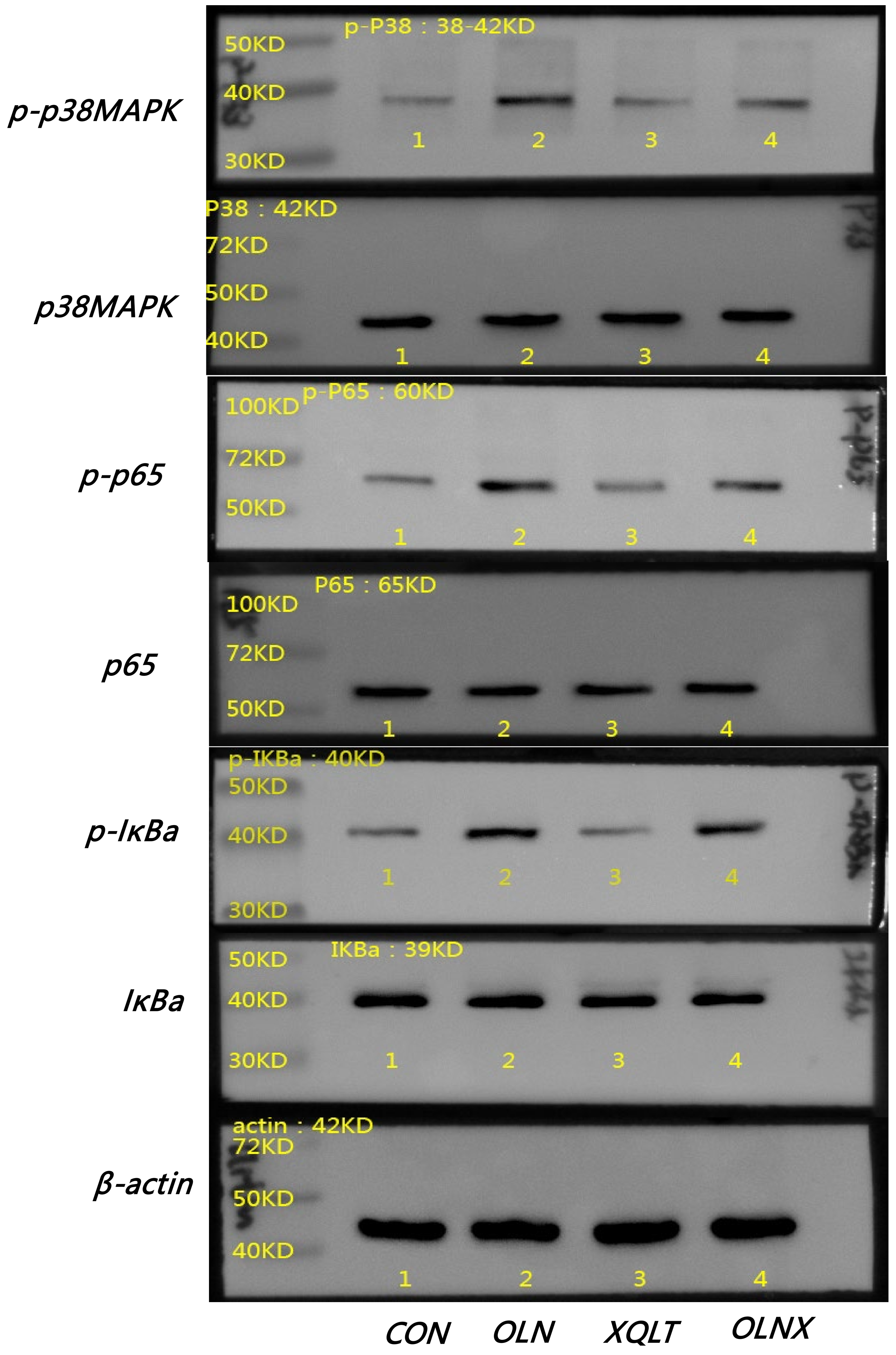

2. original image of Flow Cytometry

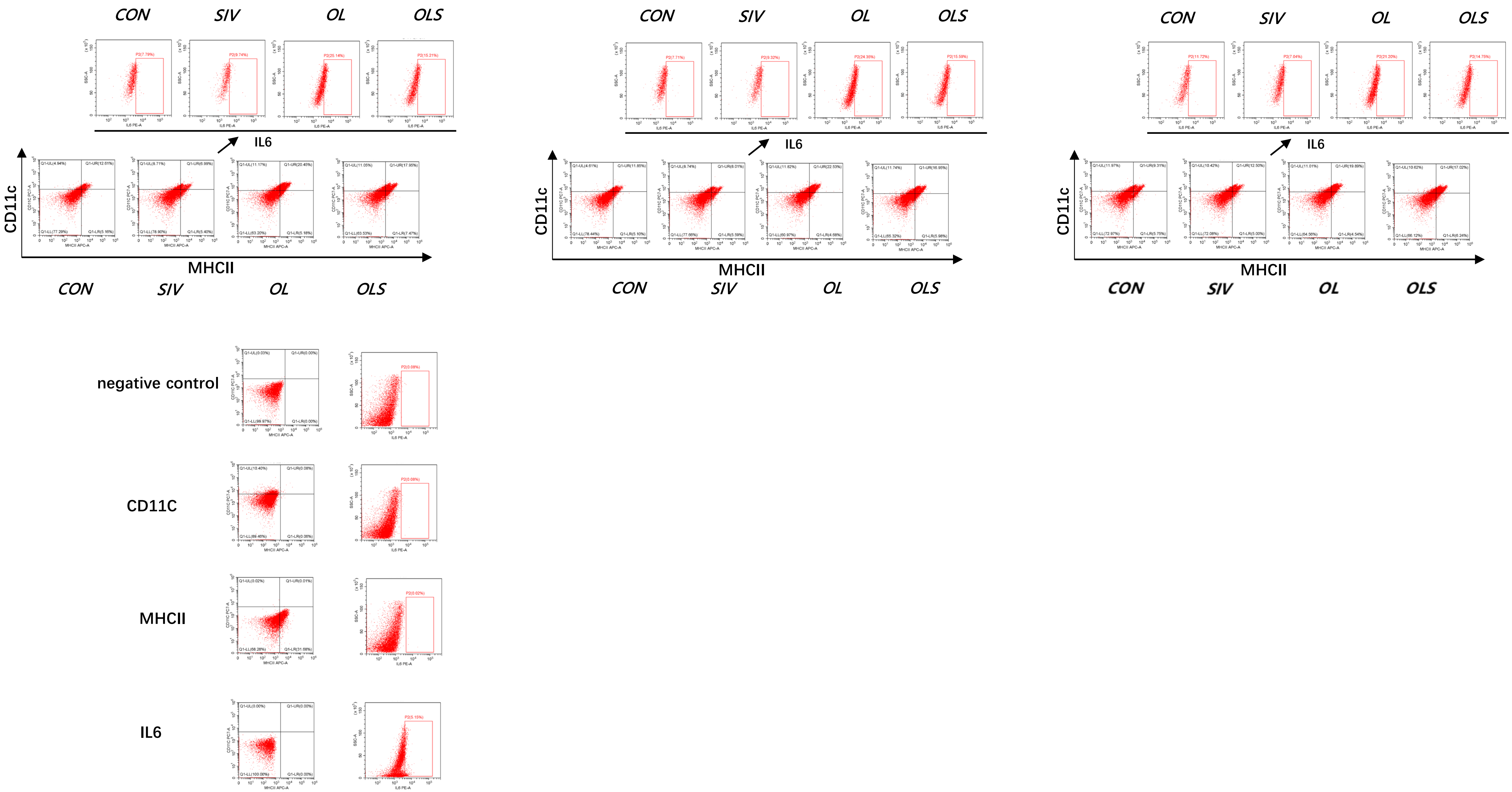
CON
SIV
OL
OLS
IL6
CD11c
MHCII
CON
SIV
OL
OLS
IL6
CD11c
MHCII
CON
SIV
OL
OLS
IL6
CD11c
MHCII
CON
SIV
OL
OLS
CON
SIV
OL
OLS
CON
SIV
OL
OLS
negative control
CD11C
MHCII
IL6

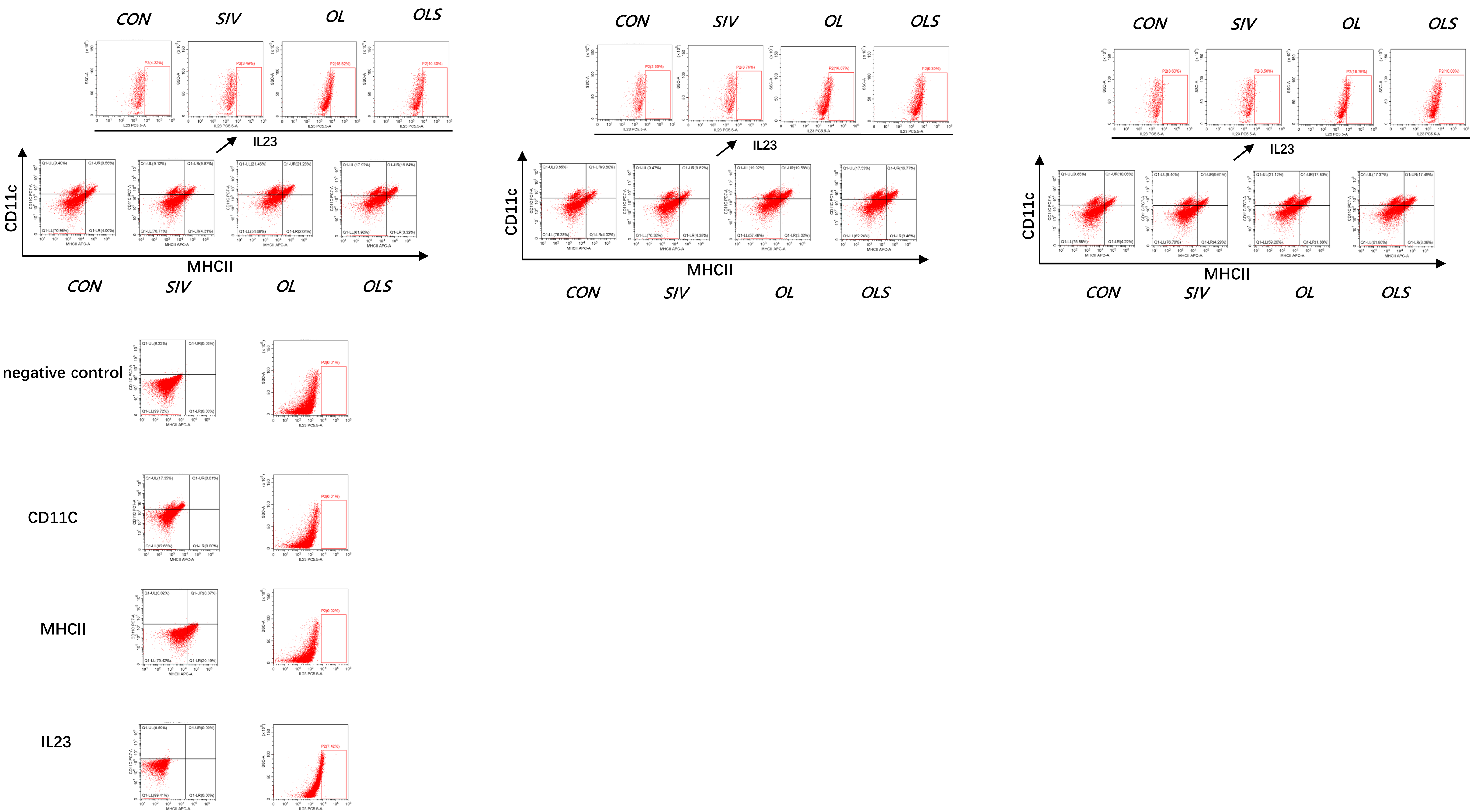

CON
SIV
OL
OLS
IL23
CD11c
MHCII
CON
SIV
OL
OLS
CON
SIV
OL
OLS
IL23
CD11c
MHCII
CON
SIV
OL
OLS
CON
SIV
OL
OLS
IL23
CD11c
MHCII
CON
SIV
OL
OLS
negative control
CD11C
MHCII
IL23

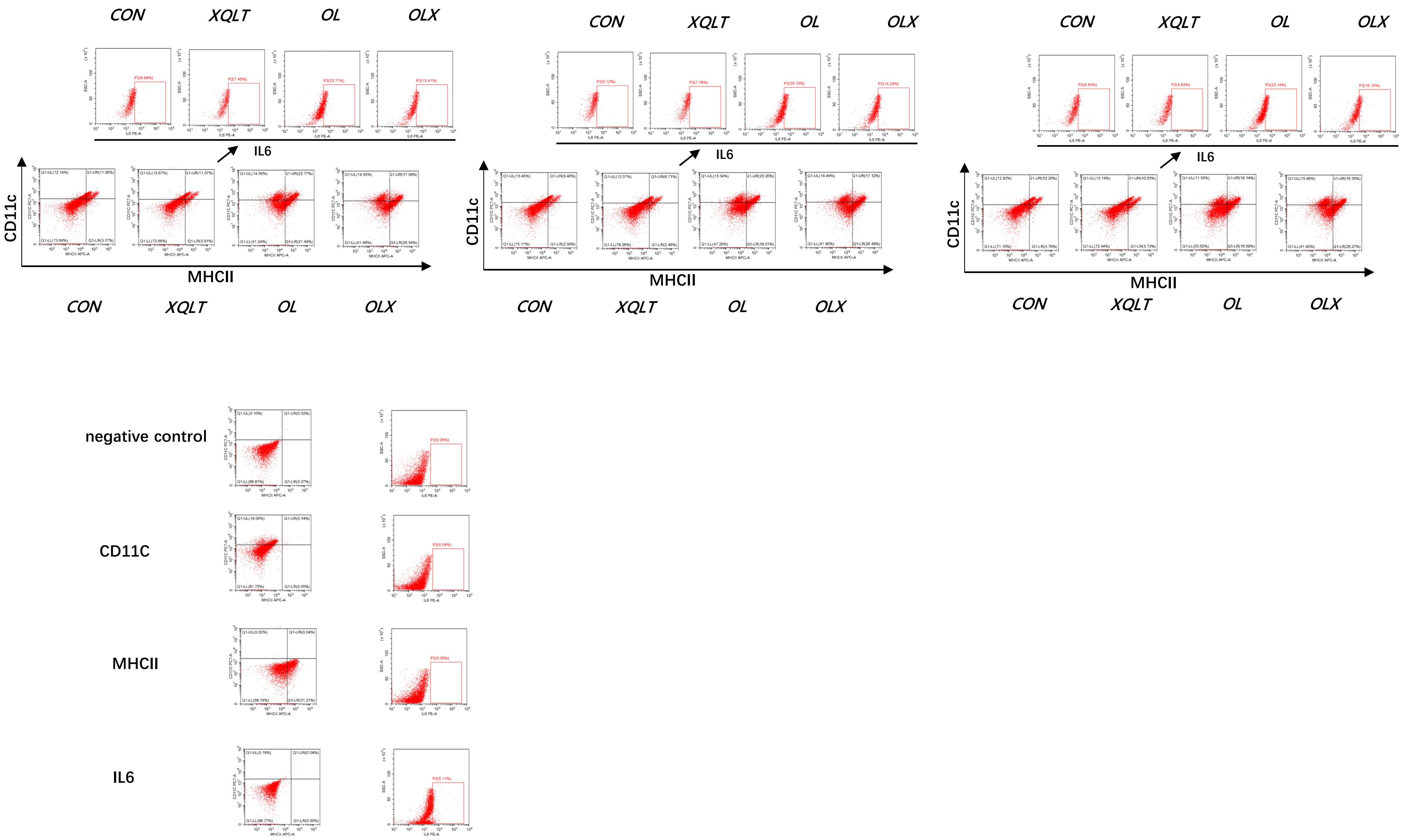
CON
XQLT
OL
OLX
IL6
CD11c
MHCII
CON
XQLT
OL
OLX
negative control
CD11C
MHCII
IL6

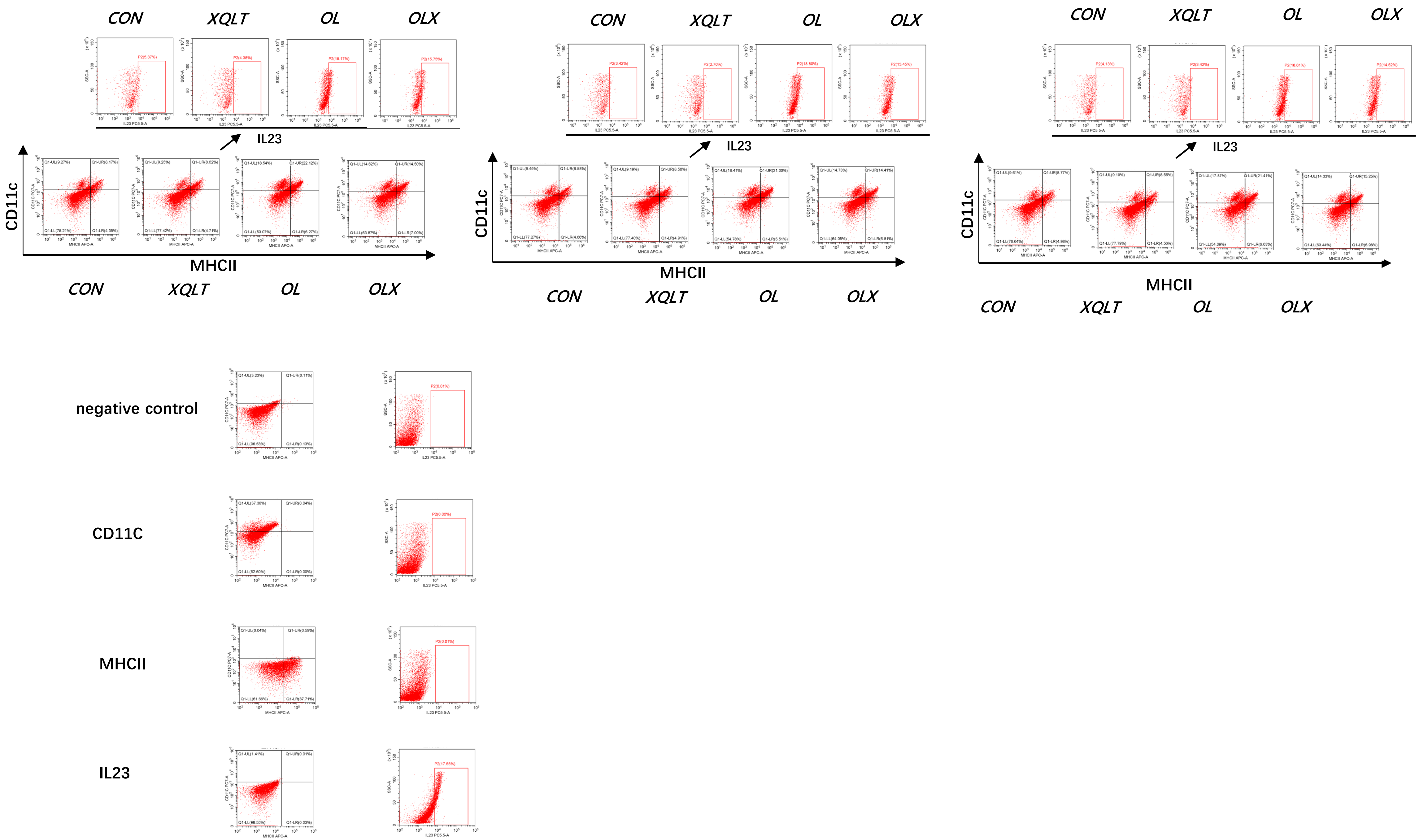
CON
XQLT
OL
OLX
IL23
CD11c
MHCII
CON
XQLT
OL
OLX
negative control
CD11C
MHCII
IL23

Figure2d

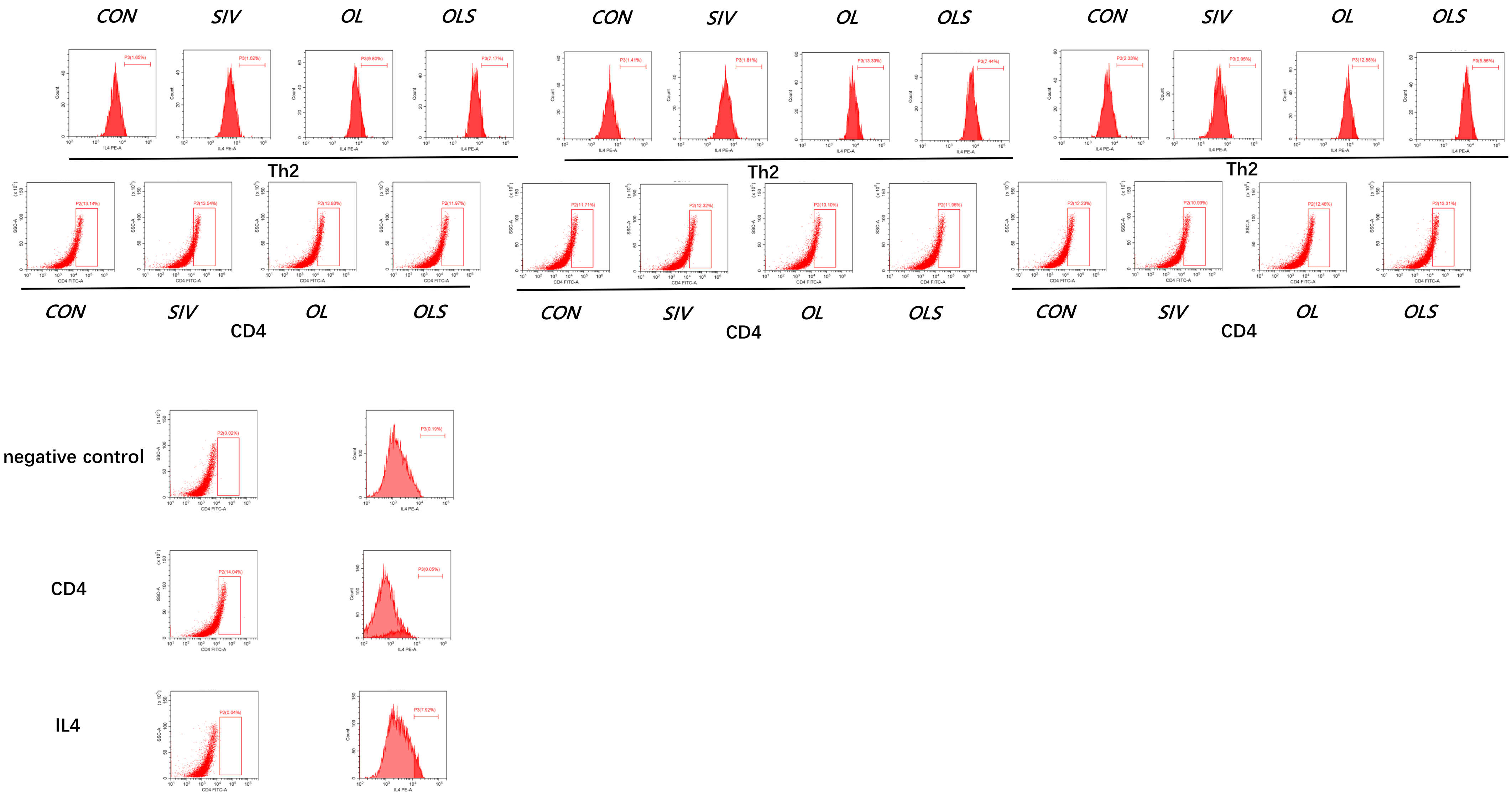

CON
SIV
OL
OLS
CON
SIV
OL
OLS
CON
SIV
OL
OLS
Th2
Th2
Th2
CON
SIV
OL
OLS
CD4
CON
SIV
OL
OLS
CD4
CON
SIV
OL
OLS
CD4
negative control
CD4
IL4

Figure2d

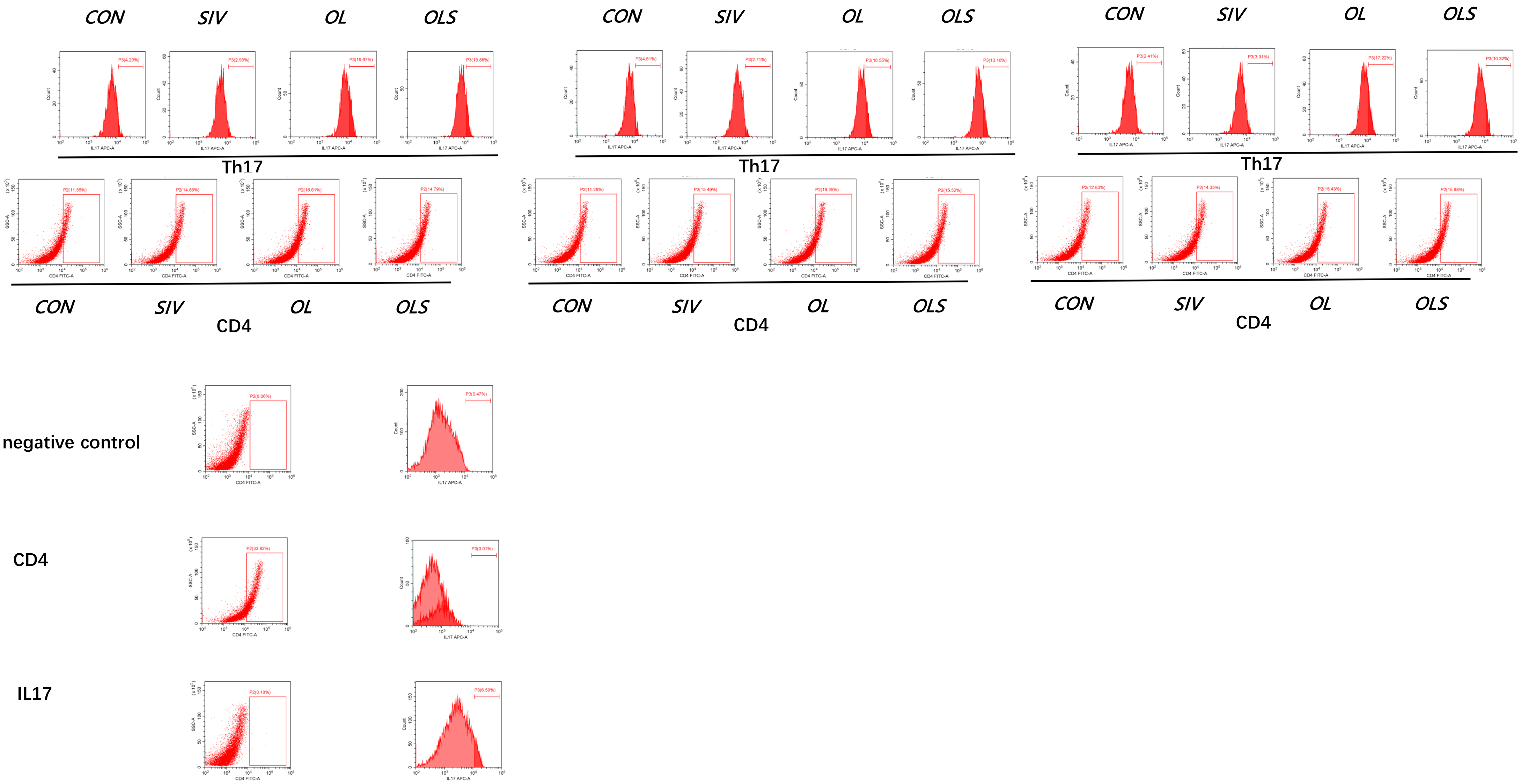
CON
SIV
OL
OLS
Th17
CON
SIV
OL
OLS
CD4
negative control
CD4
IL17

Figure4d

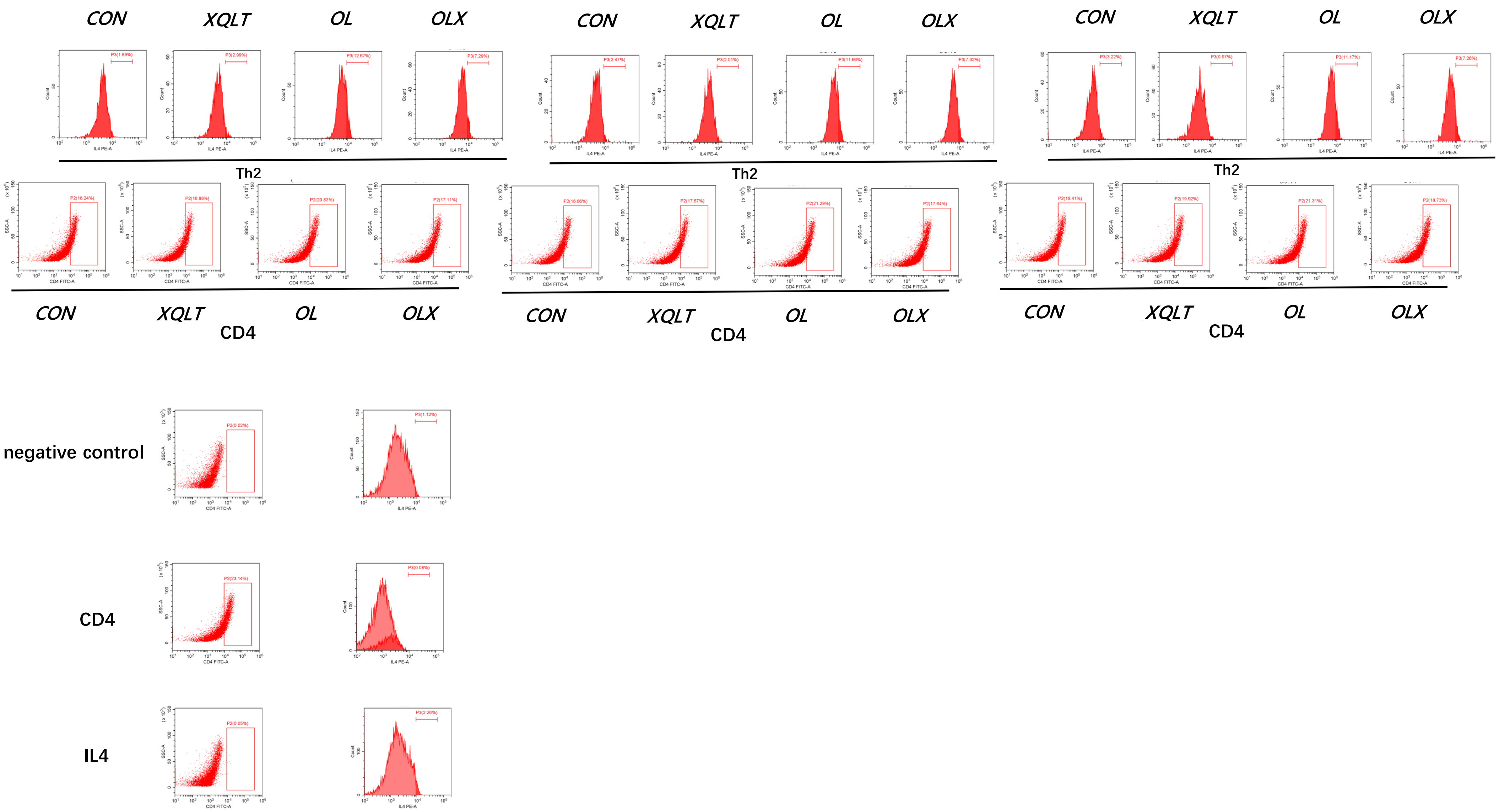

Figure4d

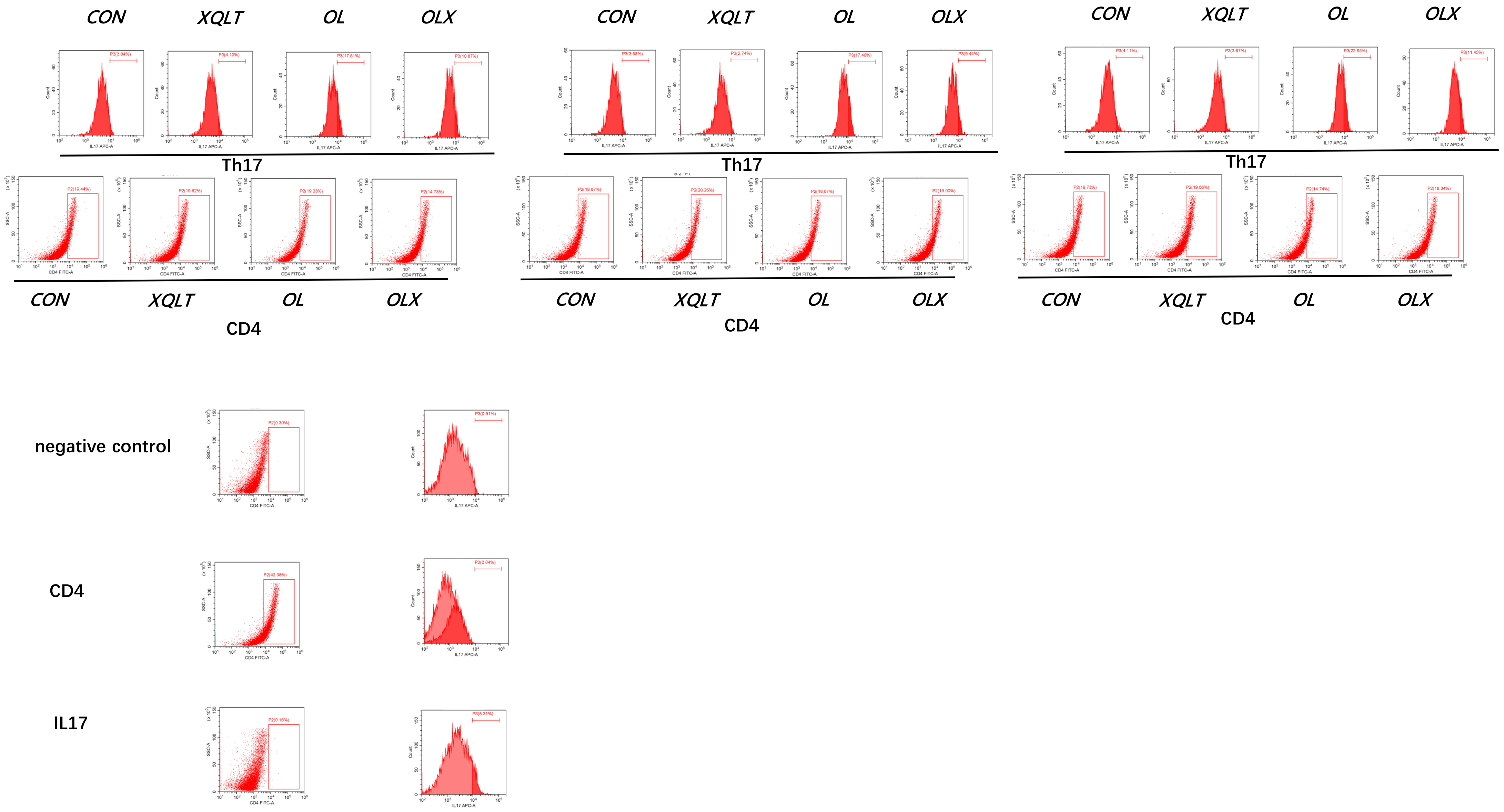

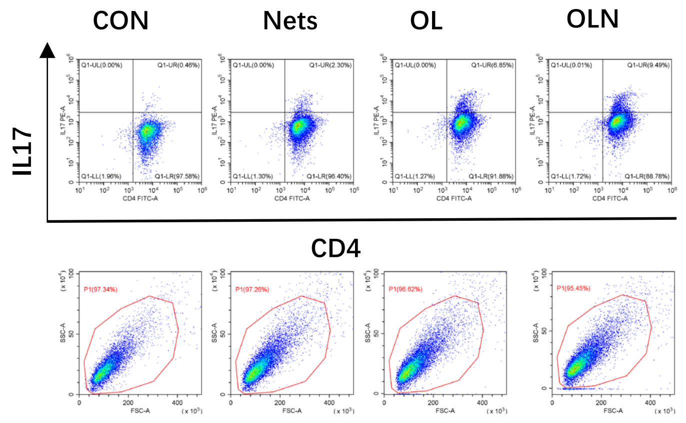
CON
Nets
OL
OLN
IL17
CD4

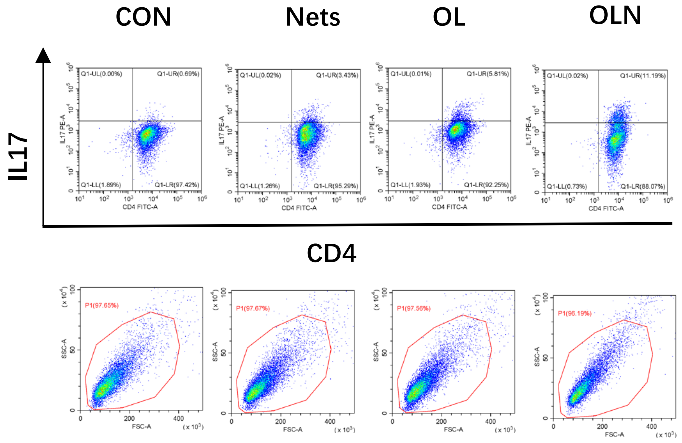
CON
Nets
OL
OLN
IL17
CD4

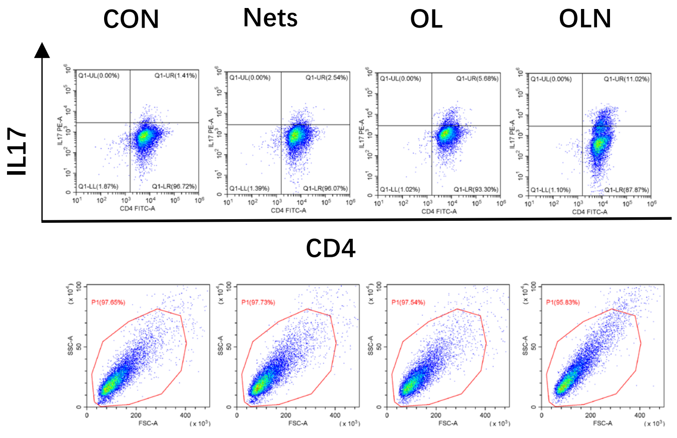
CON
Nets
OL
OLN
IL17
CD4

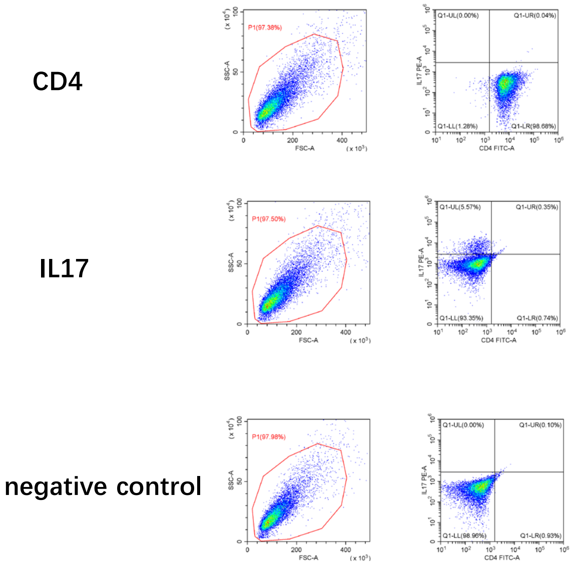
CD4
IL17
negative control

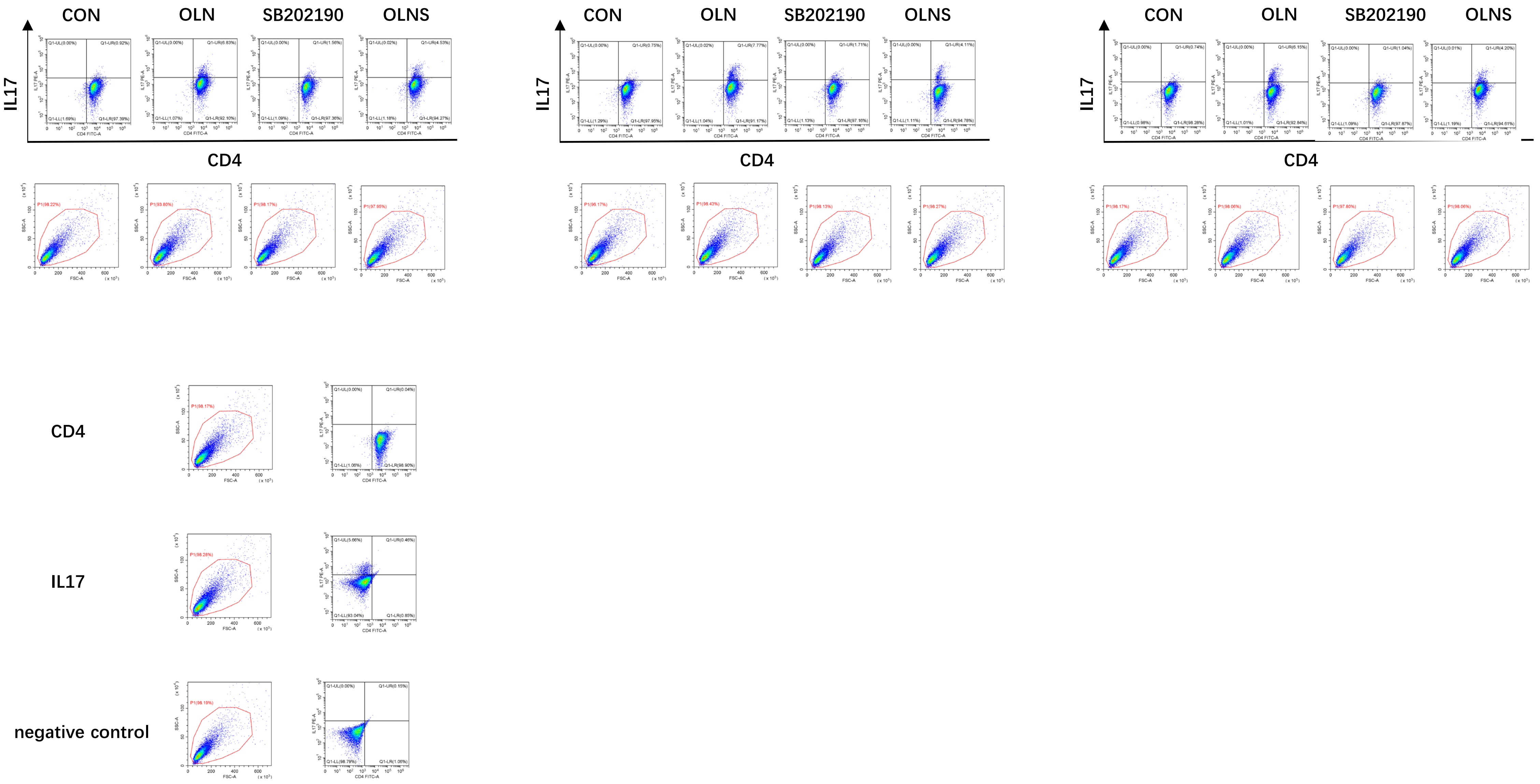

CON
OLN
SB202190
OLNS
IL17
CD4
CON
OLN
SB202190
OLNS
IL17
CD4
CON
OLN
SB202190
OLNS
IL17
CD4
CD4
IL17
negative control

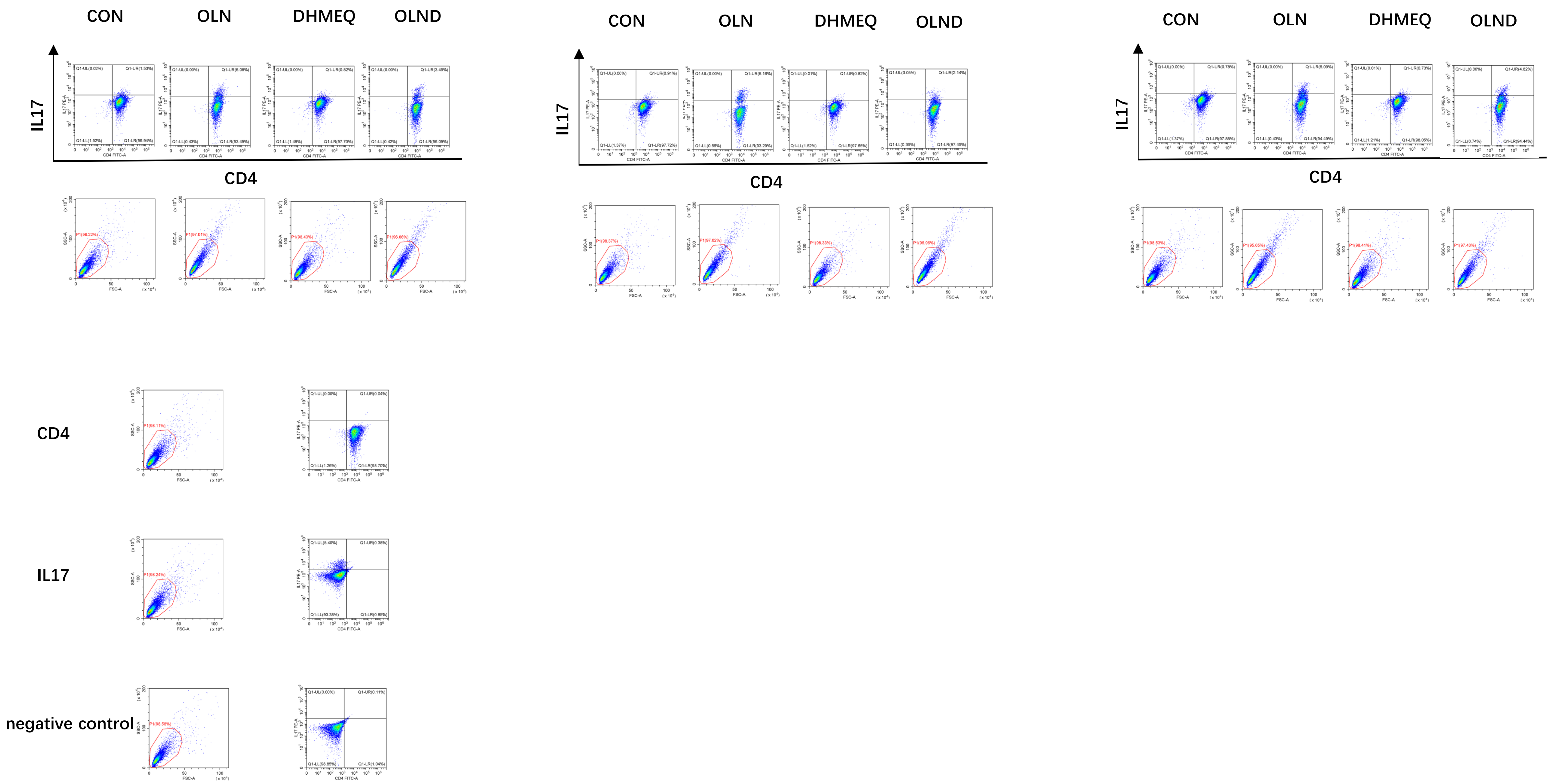
CON
OLN
DHMEQ
OLND
IL17
CD4
CON
OLN
DHMEQ
OLND
IL17
CD4
CON
OLN
DHMEQ
OLND
IL17
CD4
CD4
IL17
negative control

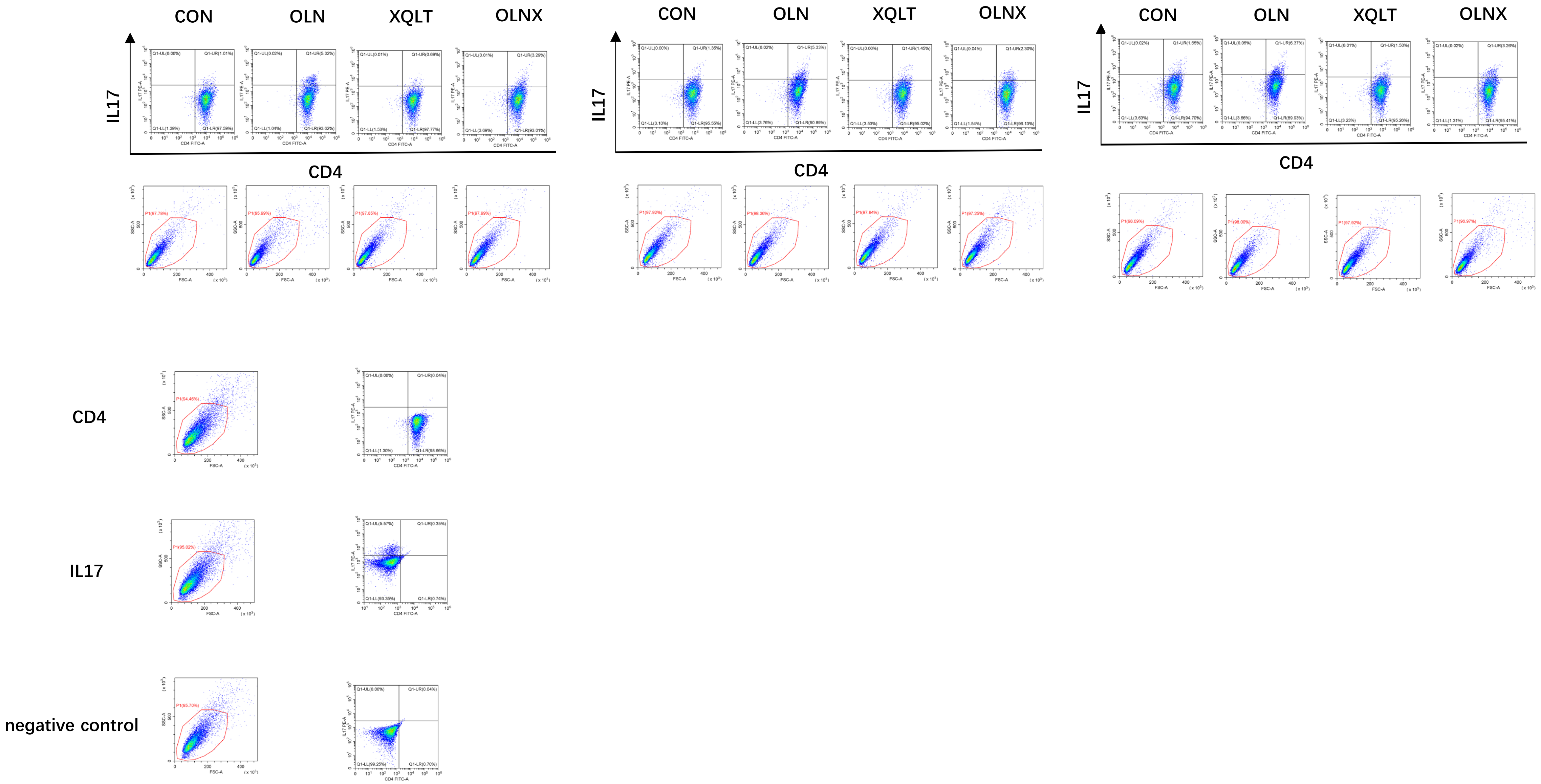

CON
OLN
XQLT
OLNX
IL17
CD4
CON
OLN
XQLT
OLNX
IL17
CD4
CON
OLN
XQLT
OLNX
IL17
CD4
CD4
IL17
negative control
